# *Melanospora* (Sordariomycetes, Ascomycota) and its relatives

**DOI:** 10.3897/mycokeys.44.29742

**Published:** 2018-12-18

**Authors:** Yasmina Marin-Felix, Josep Guarro, José F. ano-Lira, Dania García, Andrew N. iller, Alberto M. Stchigel

**Affiliations:** 1 Mycology Unit, Medical School and IISPV, Universitat Rovira i Virgili, C/ Sant Llorenç 21, 43201 Reus, Tarragona, Spain Universitat Rovira i Virgili Reus Spain; 2 Westerdijk Fungal Biodiversity Institute, P.O. Box 85167, 3508 CT Utrecht, Netherlands Westerdijk Fungal Biodiversity Institute Utrecht Netherlands; 3 Illinois Natural History Survey, University of Illinois, 1816 S. Oak St., Champaign, Illinois, USA 61820 University of Illinois Champaign United States of America

**Keywords:** Ceratostomataceae, *
Dactylidispora
*, *
Echinusitheca
*, Melanosporales, *
Microthecium
*, *
Pseudomicrothecium
*, soil, *
Sphaerodes
*, 4 new taxa

## Abstract

The order Melanosporales comprises a large group of ascomycetes, most of them mycoparasites, characterized by the production of usually ostiolate, translucent ascomata, unitunicate asci, and unicellular, pigmented ascospores with germ pores or germ slits. The most studied taxa are *Melanospora* and *Sphaerodes*, but the boundaries with other morphologically closely related genera are not well resolved. In this study, the taxonomy of *Melanospora* and related taxa have been re-evaluated based on the analysis of nuclear rDNA, actin and elongation factor genes sequences of fresh isolates and numerous type and reference strains. The genus *Melanospora* has been restricted to species with ostiolate ascoma whose neck is composed of intermixed hyphae, and with a phialidic asexual morph. *Microthecium* has been re-established for species of *Melanospora* and *Sphaerodes* without a typical ascomatal neck or, if present, being short and composed of angular cells similar to those of the ascomatal wall, and usually producing bulbils. Three new genera have been proposed: *Dactylidispora*, possessing ascospores with a raised rim surrounding both terminal germ pores; *Echinusitheca*, with densely setose, dark ascomata; and *Pseudomicrothecium*, characterized by ascospores with indistinct germ pores. Dichotomous keys to identify the accepted genera of the Melanosporales, and keys to discriminate among the species of *Melanospora* and *Microthecium*, as well as a brief description of the accepted species of both genera, are also provided.

## Introduction

The family Ceratostomataceae (Winter 1887) includes nearly 100 species, often mycoparasitic and characterized by ostiolate and rostrate, or less frequently non-ostiolate, translucent ascomata, unitunicate and evanescent asci, brown or exceptionally hyaline, unicellular ascospores with a germ pore at each end, or less frequently with only one germ pore or a germ slit, and phialidic asexual morphs or bulbils. Currently, that family is included in the Melanosporales (Chaudhary et al. 2006, Zhang et al. 2006, Hibbett et al. 2007, Li et al. 2016, Schultes et al. 2017), although historically it had been placed in Aspergillales (Gaümann 1964), Hypocreales (Alexopoulos 1962, Spatafora and Blackwell 1994a, Rehner and Samuels 1995, Jones and Blackwell 1998, Zhang and Blackwell 2002) and Sphaeriales (Bessey 1950, Dennis 1968). This family comprises 11 sexually reproducing genera, i.e. *Arxiomyces*, *Melanospora*, *Persiciospora*, *Pteridiosperma*, *Pustulipora*, *Rhytidospora*, *Scopinella*, *Setiferotheca*, *Sphaerodes*, *Syspastospora* and *Vittatispora. Melanospora*, the largest genus of this family (more than 50 species), was established by Corda (1837) to accommodate *Ceratostomachionea* and two new species, *Melanosporazamiae* and *Melanosporaleucotricha*, with the former chosen later as the type species (Kowalski 1965). *Melanospora* is characterized by usually ostiolate ascomata with a long neck and a translucent, pale yellow to reddish brown ascomatal wall, and mostly smooth-walled, brown, ellipsoidal to citriform, rarely discoid or fusoid ascospores, with a depressed germ pore at each end, occasionally surrounded by a raised rim (Guarro et al. 2012). Related genera are *Microthecium* and *Sphaerodes*. The former was erected by Corda (1842) to distinguish *Mi.zobelii* from *Melanospora* spp. by the presence of non-ostiolate, usually immersed ascomata; and *Sphaerodes* was introduced by Clements (1909) to separate *Melanosporaepisphaeria* from *Melanospora* spp. by its reticulate ascospores. However, the generic boundaries between *Melanospora* and its relatives remained obscure. Doguet (1955) carried out a revision of *Melanospora*, synonymizing several species and transferring additional species from other genera, mostly from *Sphaeroderma*, which had been proposed by Fuckel (1877) and distinguished from *Melanospora* by the absence of an ascomatal neck. Doguet (1955) considered the production of a neck as a non-stable taxonomic character influenced by the nature of the substrate where the fungus grows, and segregated the genus in several sections on the basis of the morphology of the ascomata (presence or absence of neck, and its size when present) and ascospores (shape and ornamentation). The most comprehensive revision of *Melanospora* and related genera was carried out by Cannon and Hawksworth (1982), based mainly on the structure of the ascospore wall under SEM, resulting in the transfer of species of *Microthecium* to *Melanospora* and to *Sphaerodes*. However, recent molecular studies demonstrated that these two latter genera are polyphyletic (Zhang and Blackwell 2002, Fan et al. 2012, Li et al. 2016, Schultes et al. 2017). Other genera included in the family are: *Arxiomyces*, which produces ovoid to ellipsoidal ascospores with a rounded apex and a truncate base with a large sunken germ pore (Cannon and Hawksworth 1982, 1983); *Persiciospora*, characterized by ascospores with a pitted wall and a faint reticulation (Cannon and Hawksworth 1982); *Pteridiosperma*, with ascospores ornamented with longitudinal wing-like appendages (Krug and Jeng 1979); *Pustulipora*, distinguished by its ascospores with a germ pore at each end surrounded by a blistered, rarely cushion-like structure showing an irregular pustulate appearance (Cannon 1982); *Rhytidospora*, characterized by non-ostiolate ascomata with a cephalothecoid ascomatal wall (Krug and Jeng 1979); *Scopinella*, producing brown, cuboid-ellipsoidal ascospores with two prominent longitudinal germ slits (Cannon and Hawksworth 1982); *Setiferotheca*, which produces ascospores similar to those of *Arxiomyces* and ascomata with a crown of dark brown setae surrounding the ostiole (Matsushima 1995); *Syspastospora*, possessing ascomata with a long neck composed of parallel arranged hyphae and cylindrical ascospores with a large terminal slightly sunken germ pore at each end (Cannon and Hawksworth 1982); and *Vittatispora*, which produces ascomata similar to those of *Syspastospora* and citriform ascospores with a longitudinal, thick, hyaline ridge (Chaudhary et al. 2006). Practically all taxonomic studies on these fungi have been based exclusively on the morphological characterization of the reproductive structures of preserved fungarium specimens, since unfortunately due to their mycoparasitism, many of these fungi do not grow in pure culture or do not produce ascomata in absence of their hosts. On the other hand, obtaining reliable nucleotide sequences from members of the Melanosporales is also difficult because of the usually large amount of DNA of their hosts. Based on the study of several freshly-isolated soil-borne fungi and of reference and type strains obtained from various culture collections, we have re-examined the phylogenetic relationships of the most relevant genera of the Ceratostomataceae. Consequently, the genus *Melanospora* has been redefined, *Microthecium* has been re-established, and three new genera have been proposed.

## Materials and methods

### Fungal isolates

The strains included in this study are listed in Table 1. Fresh isolates were obtained from samples following previously described procedures for the activation of dormant ascospores in soil using acetic acid and phenol solutions (Stchigel et al. 2001, García et al. 2003). Ascomata were transferred to 55 mm diam. Petri dishes containing oatmeal agar (OA; oatmeal flakes, 30 g; agar-agar, 20 g; distilled water, 1 L) using a sterile needle, which were then incubated at 15, 25 and 35 °C.

### Morphological study

For cultural characterization, isolates were grown for up to 30 d on OA, potato carrot agar (PCA; grated potatoes, 20 g; grated carrot, 20 g; agar-agar, 20 g; L-chloramphenicol, 100 mg; distilled water, 1 L), and potato dextrose agar (PDA; Pronadisa, Madrid, Spain) at 5, 10, 15, 20, 25, 30, 35 and 40 °C. Color notations in parentheses are from Kornerup and Wanscher (1984). Vegetative and reproductive structures were examined under an Olympus BH-1 brightfield microscope by direct mounting in lactic acid and water of the ascomata and/or microcultures grown on OA and PDA. Pictures were obtained with a Zeiss Axio Imager M1 brightfield microscope. The samples for scanning electron microscopy (SEM) were processed according to Figueras and Guarro (1988), and SEM micrographs were taken at 15 keV with a Jeol JSM 840 microscope.

### Molecular study

**Figure 1.**
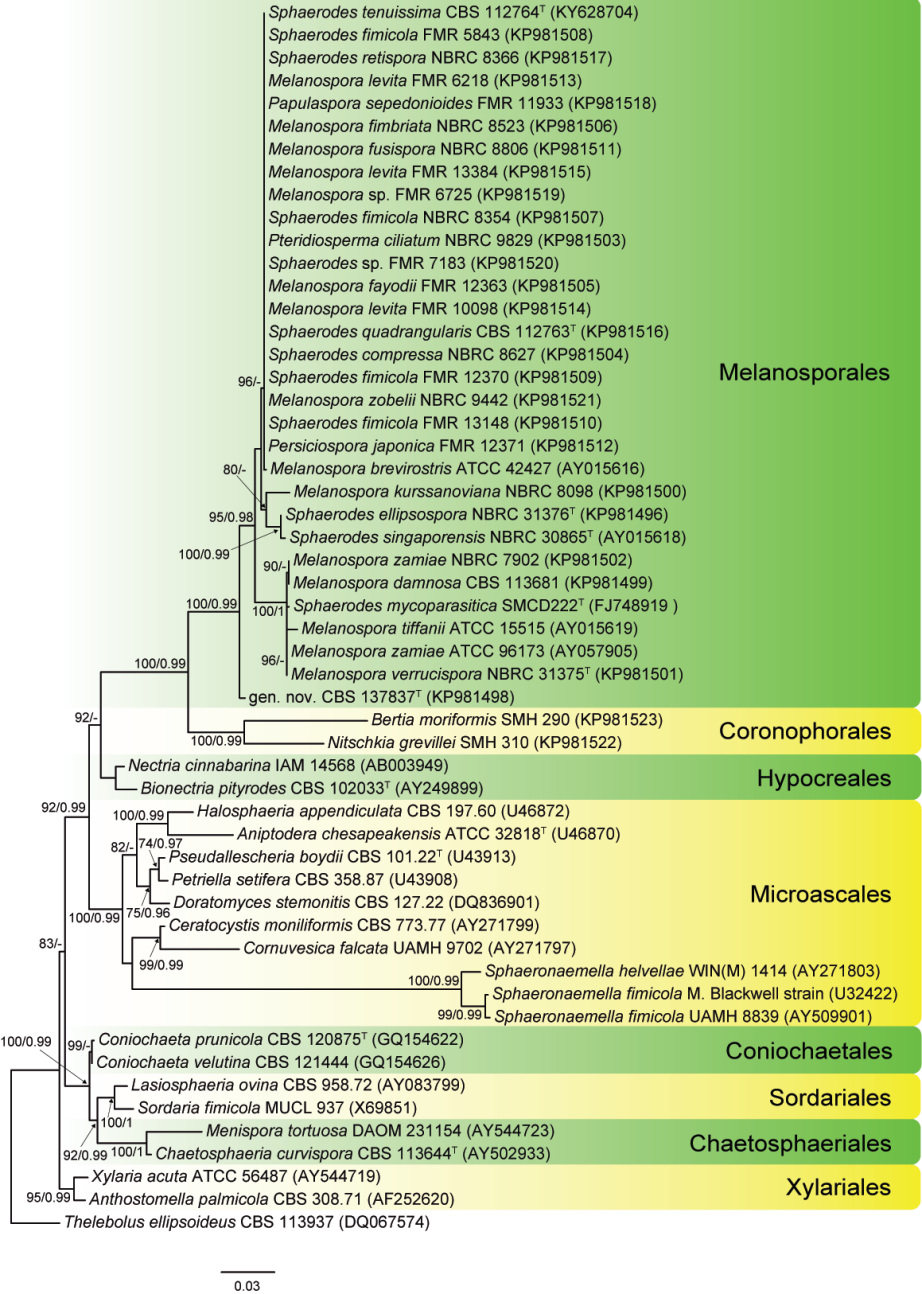
RAxML phylogram obtained from SSU sequences of isolates and type and reference strains included in the Melanosporales, and strains belonging to the orders Chaetosphaeriales, Coniochaetales, Coronophorales, Hypocreales, Microascales, Sordariales and Xylariales. *Thelebolusellipsoideus* was used as outgroup. RAxML bootstrap support (BS) values above 70 % and Bayesian posterior probability scores above 0.95 are shown at the nodes. Type strains of the different species are indicated with ^T^.

The DNA of the fungal isolates (Table 1) was extracted and purified directly from the colonies according to the Fast DNA Kit protocol (MP Biomedicals, Solon, Ohio). The amplification of the small subunit (SSU), the D1−D3 domains of the large subunit (LSU) and the internal transcribed spacer region (ITS) of the nuclear rDNA, and the fragments of actin (*act*) and translation elongation factor 1-α (*tef1*) genes were performed according to White et al. (1990) (SSU), Vilgalys and Hester (1990) (LSU), Cano et al. (2004) (ITS), Voigt and Wöstermeyer (2000) (*act*) and Houbraken et al. (2007) (*tef1*). A BigDye Terminator 3.1 cycle sequencing kit (Applied Biosystems Inc., Foster City, California) was used to sequence both strands with a combination of the same primers used in the amplification. PCR products were purified and sequenced at Macrogen Europe (Amsterdam, The Netherlands) with a 3730XL DNA analyzer (Applied Biosystems), and the consensus sequences were obtained using SeqMan (version 7.0.0; DNASTAR, Madison, WI, USA). A phylogenetic study based on the analysis of SSU sequences of the isolates and type and reference strains of the Melanosporales and of some members of the Chaetosphaeriales, Coniochaetales, Coronophorales, Hypocreales, Microascales, Sordariales and Xylariales, using *Thelebolusellipsoideus* (Thelebolales) as outgroup, was performed to confirm the taxonomic placement of our isolates. A subsequent study, carried out to infer the phylogenetic relationships among members of the Melanosporales, was based on the analysis of a combined data set including the ITS, LSU, *act* and *tef1* sequences of our isolates and of type and reference strains of a large number of the Melanosporales, including *Nectriacinnabarina* and *Pseudallescheriafusoidea* as outgroups. The Maximum-Likelihood (ML) and Bayesian Inference (BI) methods were used in phylogenetic analyses as described by Hernández-Restrepo et al. (2016). Bootstrap support (BS) ≥70 and posterior probability values (PP) ≥0.95 were considered significant. The sequences generated in this study were deposited in GenBank (Table 1 and Fig. 1) and the alignments used in the phylogenetic analyses were deposited in TreeBASE (http://purl.org/phylo/treebase/phylows/study/TB2:S17079). Sequences retrieved from GenBank and NBRC included in the SSU and combined analyses are shown in Fig. 1 and Table 1, respectively.

**Table 1.** Isolates and reference strains of members of Melanosporales included in the combined phylogenetic study.

Taxa	Strain	Source	GenBank accession number			
			LSU	ITS	*act*	*tef1*
* Dactylidispora ellipsospora *	NBRC 31376^T^	Forest soil, Papua New Guinea, Buin, Bougainville Island	KP981451	03137601*	KP981545	KP981579
* Dactylidispora singaporensis *	NBRC 30865^T^	Soil, Singapore	KP981452	03086502*	KP981546	KP981580
* Echinusitheca citrispora *	CBS 137837^T^ = FMR 12767^T^	Forest soil, USA, North Carolina, Great Smoky Mountains National Park, Cataloochee Creek Campground	KP981453	KP981477	KP981547	KP981581
* Nectria cinnabarina *	CBS 127383	Austria, Niederösterreich, Litschau	HM534894	HM534894	–	HM534873
* Melanospora damnosa *	CBS 113681	Soil, France, Pont d’Espagne	KP981454	KP981478	KP981543	KP981582
* Melanospora kurssanoviana *	NBRC 8098	Unknown	KP981455	KP981479	KP981548	KP981583
* Melanospora verrucispora *	NBRC 31375^T^	Forest soil, Papua New Guinea, Kebil, Chimb Dist.	KP981456	KP981480	KP981549	KP981584
* Melanospora zamiae *	NBRC 7902	Unknown	KP981457	00790201*	KP981544	KP981585
* Microthecium ciliatum *	NBRC 9829	Soil, unknown	KP981458	KP981481	KP981524	KP981586
* Microthecium compressum *	NBRC 8627	Unkown	KP981459	00862701*	KP981525	KP981587
* Microthecium fayodii *	FMR 12363	Soil, Tennessee, Great Smoky Mountains National Park, Cosby Creek trail	KP981460	KP981482	KP981526	KP981588
* Microthecium fimbriatum *	NBRC 8523	Unknown	KP981461	KP981483	KP981527	KP981589
* Microthecium fimicola *	NBRC 8354	Unknown	KP981462	KP981484	KP981528	KP981590
	FMR 5483	Soil, Australia, Moara	KP981463	KP981485	KP981529	KP981591
	FMR 12370	Soil, Spain, Gran Canaria	KP981464	KP981486	KP981530	KP981592
	FMR 13418	Soil, Spain, Aragon, Los Valles Occidentales	KP981465	KP981487	KP981531	KP981593
* Microthecium fusisporum *	NBRC 8806	Unknown	KP981466	00880601*	KP981532	KP981594
* Microthecium japonicum *	FMR 12371	Soil, Spain, Gran Canaria, Pico de Osorio	KP981467	KP981488	KP981533	KP981595
* Microthecium levitum *	FMR 6218 = CBS 966.97	Soil, Nepal, Bhadgaon	KP981468	KP981489	KP981534	KP981596
	FMR 10098	Soil, Nigeria, Enugu, Nsukka	KP981469	KP981490	KP981535	KP981597
	FMR 13884	Soil, Spain, Catalonia, Vall Fosca	KP981470	KP981491	KP981536	KP981598
* Microthecium quadrangulatum *	CBS 112763^T^	Soil, Spain, Asturias, Muniellos Biological Absolute Reserve	KP981471	KP981492	KP981537	KP981599
* Microthecium retisporum *	NBRC 8366	Soil, Japan	KP981472	00836601*	KP981538	KP981600
* Microthecium sepedonioides *	FMR 11933	Forest soil, Spain, Aragón, valle de Ordesa	KP981473	KP981493	KP981539	KP981601
*Microthecium* sp.	FMR 6725 = CBS 102190	Desert soil, Egypt, Sinai	KP981474	KP981494	KP981540	KP981602
*Microthecium* sp.	FMR 7183 = CBS 108937	Forest soil, New South Wales, Sydney, Blue Mountains	KP981475	KP981495	KP981541	KP981603
* Microthecium tenuissimum *	CBS 112764^T^	Soil, Spain, Murcia, Sierra de Espuña, Umbria de Peña Apartada	KY628706	KY628705	–	–
* Microthecium zobelii *	NBRC 9442	Decaying carpophore of *Coriolusflabelliformis*	KP981476	00944201*	KP981542	KP981604
* Pseudallescheria fusoidea *	CBS 106.53^T^	Soil, Panama, Guipo	EF151316	AY878941	–	–
* Pseudomicrothecium subterraneum *	BJTC FAN1001^T^	From *Tuberindicum*, China, Yunnan	JN247804	–	–	–
* Vittatispora coorgii *	BICC 7817^T^	Soil, India, Western Ghats, Coorg District, Kakkabe	DQ017375	–	–	–

BICC: Biocon culture collection, Bangalore, India; BJTC: Capital Normal University, Beijing, China; CBS: Westerdijk Fungal Biodiversity Institute, Utrecht, the Netherlands; FMR: Facultat de Medicina, Reus, Spain; NBRC: Biological Resource Center, Chiba, Japan. ^T^ zindicates type strains. * sequences retrieved from NBRC database.

## Results

The SSU phylogenetic study was based on an alignment of 1023 bp and produced a single ML tree (Fig. 1) inferred from a RAxML analysis. The members of the Melanosporales including our isolates were placed in a highly supported main clade (100 % BS / 0.99 PP), and the isolate CBS 137837, whose morphological features did not match any previously described taxon, occurred as a basal branch clearly separated from the other Melanosporales, which grouped together with a high support (95 % BS / 0.98 PP) and separated into three subclades. The first one (96 % BS / - PP), contained most of the isolates morphologically identified as *Melanospora*, *Persiciospora* and *Sphaerodes*, including the type and reference strains of *Melanosporabrevirostris*, *M.fimbriata*, *M.fusispora*, *M.levita*, *M.zobelii*, *Papulasporasepedonioides*, *Pteridiospermaciliatum*, *Sphaerodescompressa*, *S.fimicola*, *S.retispora*, *S.quadrangularis* and *S.tenuissima*, without significant genetic variation among them. The second subclade (80 % BS / - PP) comprised the type strains of *Sphaerodesellipsospora* and *Sphaerodessingaporensis* and a reference strain of *Melanosporakurssanoviana*, which resulted clearly separated from the other two, which grouped with high support (100 % BS / 0.98 PP). In the third subclade (100 % BS / 1 PP) were nested the type species of *Melanospora* (*M.zamiae*), the type strains of *Melanosporaverrucispora* and *Sphaerodesmycoparasitica*, and reference strains of *Melanosporadamnosa* and *Melanosporatiffanii*.

**Figure 2.**
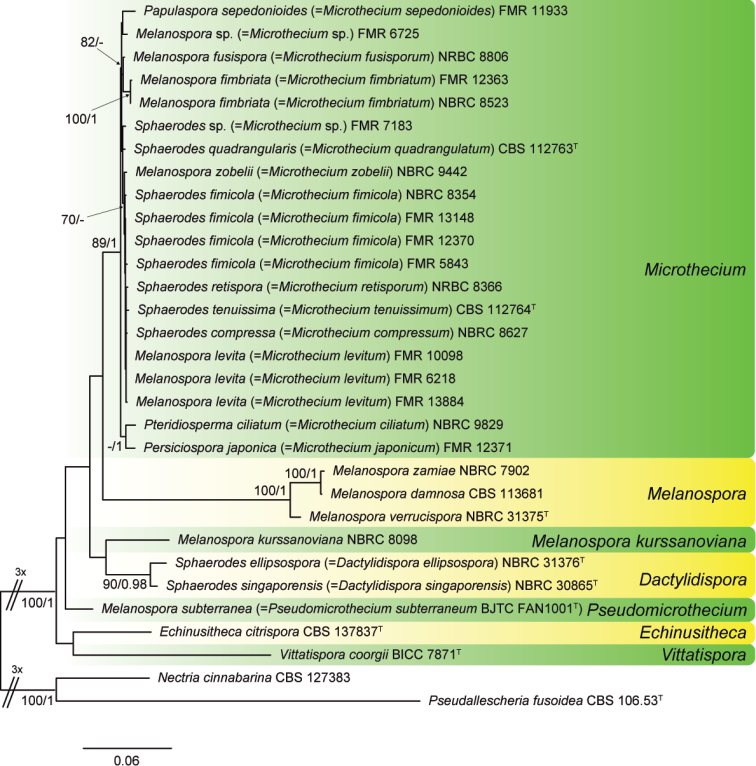
RAxML phylogram obtained from the combined ITS, LSU, *act* and *tef1* sequences of our isolates and type and reference strains of the order Melanosporales. *Nectriacinnabarina* and *Pseudallescheriafusoidea* were used as outgroup. RAxML bootstrap support (BS) values above 70 % and Bayesian posterior probability scores above 0.95 are shown at the nodes. GenBank accession numbers are indicated in Table 1. Type strains of the different species are indicated with ^T^.

The lengths of the individual alignments used in the combined data set were 802 bp (LSU), 535 bp (ITS), 727 bp (*act*) and 846 bp (*tef1*), respectively, and the final total alignment was 2910 bp. In the ML tree derived from the RAxML analysis of the combined data set (Fig. 2), the Melanosporales were highly supported (100 % BS / 1 PP) and subdivided into seven lineages. The first clade (89 % BS / 1 PP; Clade *Microthecium*) grouped all our isolates, with the exception of CBS 137837, and type or reference strains of *Melanosporafimbriata*, *M.fusispora*, *M.levita*, *M.zobelii*, *Papulasporasepedonioides*, *Pteridiospermaciliatum*, *Sphaerodescompressa*, *S.fimicola*, *S.retispora*, *S.quadrangularis* and *S.tenuissima*. All the fungi belonging to this clade have non-ostiolate ascomata, or when a neck is present, it is short and composed of angular cells similar to those of the ascomatal wall. Also, bulbils (microsclerotial-like asexual propagules) are present in most of these species. In spite of the high morphological variability shown by members of this clade, the loci used in the phylogenetic analysis were not able to separate the species from each other. The second clade (100% BS / 1 PP; Clade *Melanospora*) comprised the type species of *Melanospora*, *M.zamiae*, the type strain of *M.verrucispora* and a reference strain of *M.damnosa*. The members of this clade produce ostiolate ascomata with a long neck composed of hyphae irregularly arranged and ending in a crown of setae. In addition, an asexual morph is commonly present, which is characterized by solitary, sessile, flask-shaped phialides producing from rounded to ellipsoidal conidia. The third lineage comprised only a reference strain of *Melanosporakurssanoviana*, which failed to sporulate in pure culture. The fourth clade (90 % BS / 0.98 PP; Clade *Dactylidispora*) was composed of the type strains of *Sphaerodesellipsospora* and *S.singaporensis*, both characterized by ascospores with a raised rim surrounding the germ pores. Finally, the isolate CBS 137837 and the type strains of *Melanosporasubterranea* and *Vittatisporacoorgii* formed three independent branches. The isolate CBS 137837 produces globose, non-ostiolate, densely setose, dark ascomata and smooth-walled ascospores with a depressed germ pore at each end, while the other two species of this clade also possess morphological features unique in the Melanosporales, e.g. ascospores with indistinct germ pores in *M.subterranea* and with a longitudinal, thick, hyaline ridge in *V.coorgii*.

### Taxonomy

#### Key to the accepted genera of the Melanosporales producing sexual morphs (adapted from Cannon and Hawksworth 1982)

**Table:** 

1	Ascospores with two longitudinal germ slits	*** Scopinella ***
–	Ascospores with germ pores	**2**
2	Ascospores with a broad germ pore and a small basal appendage	**3**
–	Ascospores with a germ pore at each end	**4**
3	Ascomata with a crown of dark brown setae surrounding the ostiole	*** Setiferotheca ***
–	Ascomata without setae	*** Arxiomyces ***
4	Ascospores oblong or cylindric-fusiform, and germ pores crateriform	*** Syspastospora ***
–	Ascospores and germ pores otherwise	**5**
5	Ascomata ostiolate; neck long, composed of hyphae	**6**
–	Ascomata non-ostiolate or ostiolate; neck absent or short, conical, composed of angular cells similar to those of the ascomatal wall	**7**
6	Neck composed of irregularly arranged hyphae	*** Melanospora ***
–	Neck composed of parallel arranged hyphae	*** Vittatispora ***
7	Ascospores with indistinct germ pores	*** Pseudomicrothecium ***
–	Ascospores with conspicuous germ pores	**8**
8	Germ pores surrounded by hyaline structures	**9**
–	Germ pores without such structures	**10**
9	Germ pores with a raised rim	*** Dactylidispora ***
–	Germ pores with a blistered, rarely cushion-like structure	*** Pustulipora ***
10	Ascomatal wall cephalothecoid	*** Rhytidospora ***
–	Ascomatal wall not cephalothecoid	**11**
11	Ascomata dark, densely setose	*** Echinusitheca ***
–	Ascomata translucent, glabrous or surrounded by hyphae-like hairs	*** Microthecium ***

##### Dactylidispora

Taxon classificationAnimaliaMelanosporalesCeratostomataceae

Y. Marín, Stchigel, Guarro & Cano gen. nov.

812079

###### Type species.

*Dactylidisporaellipsospora* (Takada) Y. Marín, Stchigel, Guarro & Cano. Holotype and ex-type strain: NBRC 31376.

###### Description.

*Ascomata* superficial, globose to pyriform, ostiolate or not, yellowish-brown, appearing dark brown when the ascospores are mature, glabrous or setose; *necks* cellular, short, conical, with a crown of setae surrounding the ostiole; *ascomatal wall* membranaceous, of *textura angularis*. *Paraphyses* absent. *Asci* 8-spored, broadly clavate, short-stipitate, without apical structures, evanescent. *Ascospores* one-celled, at first hyaline, becoming brown to dark brown when mature, fusiform or citriform, umbonate and truncate at the ends, smooth-walled, with one germ pore at each end; *germ pores* depressed, surrounded by a raised rim. *Conidiophores* reduced to conidiogenous cells. *Conidiogenous cells* phialidic, solitary, flask-shaped. *Conidia* hyaline, subglobose to ovoid, smooth-walled.

###### Etymology.

From Greek *δακτυλίδης*–, ring, and from Latin –*spora*, spore, due to the raised rim that surrounds the germ pores of the ascospores.

###### Notes.

The most distinctive characteristic of *Dactylidispora* is the production of smooth-walled ascospores with a germ pore at each end surrounded by a raised rim. *Vittatispora*, proposed as a new genus by Chaudhary et al. (2006), also produces a raised rim surrounding the germ pores. However, both genera can be easily distinguished by the nature of the ascomatal neck, which is composed of angular cells in *Dactylidispora* and of parallel arranged hyphae in *Vittatispora*; and by the presence of a hyaline ridge running the entire vertical length of the ascospore between the germ pores in *Vittatispora*. Moreover, in our phylogenetic study (Fig. 2), *Vittatispora* also constituted a lineage independent from the other members of the Melanosporales. *Pustulipora* is also morphologically similar to *Dactylidispora* being characterized by blistered, rarely cushion-like structures surrounding the germ pore (Cannon 1982). However, unfortunately, *Pustulipora* could not be included into this phylogenetic study since living cultures were not available.

The presence of a raised rim was also described in *Melanosporacollipora* (Stchigel et al. 1997), which is here transfered to *Dactylidispora* even though it was not possible to include this species in the phylogenetic study.

##### Dactylidispora collipora

Taxon classificationAnimaliaMelanosporalesCeratostomataceae

(Stchigel & Guarro) Y. Marín, Stchigel, Guarro & Cano comb. nov.

812080

Melanospora collipora Stchigel & Guarro, in Stchigel, Guarro & Figueras, Mycol. Res. 101: 446. 1997. [Basionym]

###### Notes.

This species produces ascomata with a crown of setae around the ostiole, ellipsoidal ascospores, and bulbils.

##### Dactylidispora ellipsospora

Taxon classificationAnimaliaMelanosporalesCeratostomataceae

(Takada) Y. Marín, Stchigel, Guarro & Cano comb. nov.

812081

Microthecium ellipsosporum Takada, in Kobayasi et al., Bull. natn. Sci. Mus., Tokyo 16: 527. 1973. [Basionym]
≡ Sphaerodesellipsospora (Takada) D. García, Stchigel & Guarro, Stud. Mycol. 50: 67. 2004.

###### Notes.

*Dactylidisporaellipsospora* is characterized by non-ostiolate ascomata, fusiform ascospores and absence of asexual morph.

##### Dactylidispora singaporensis

Taxon classificationAnimaliaMelanosporalesCeratostomataceae

(Morinaga, Minoura & Udagawa) Y. Marín, Stchigel, Guarro & Cano comb. nov.

812082

Melanospora singaporensis Morinaga, Minoura & Udagawa, Trans. Mycol. Soc. Japan 19: 142. 1978. [Basionym]
≡ Sphaerodessingaporensis (Morinaga, Minoura & Udagawa) D. García, Stchigel & Guarro, Stud. Mycol. 50: 67. 2004.

###### Notes.

*Dactylidisporasingaporensis* is distinguished by its ostiolate ascomata, citriform ascospores, and phialidic asexual morph.

##### Echinusitheca

Taxon classificationAnimaliaMelanosporalesCeratostomataceae

Y. Marín, Stchigel, Dania García, Guarro, A.N. Mill. & Cano gen. nov.

812084

Fig. 3

###### Type species.

*Echinusithecacitrispora* Y. Marín, Stchigel, Dania García, Guarro, A.N. Mill. & Cano. Holotype and ex-type strain, respectively: CBS H-21596, CBS 137837 = FMR 12767.

###### Description.

*Ascomata* superficial or immersed, solitary to gregarious, globose, non-ostiolate, strongly setose, semi-translucent, pale brown to brown, appearing black when ascospores are mature; *setae* straight, becoming sinuous toward apex, pale brown to brown, non-septate, rarely 1-septate, thick-walled, verrucose to tuberculate, sometimes branched; *ascomatal wall* membranaceous, of *textura angularis* to *textura globulosa*. *Asci* 8-spored, globose to subglobose, non-stipitate, without apical structures. *Ascospores* at first hyaline, becoming brown to dark brown when mature, ellipsoidal, one-celled, smooth-walled, with a depressed germ pore at each end.

**Figure 3.**
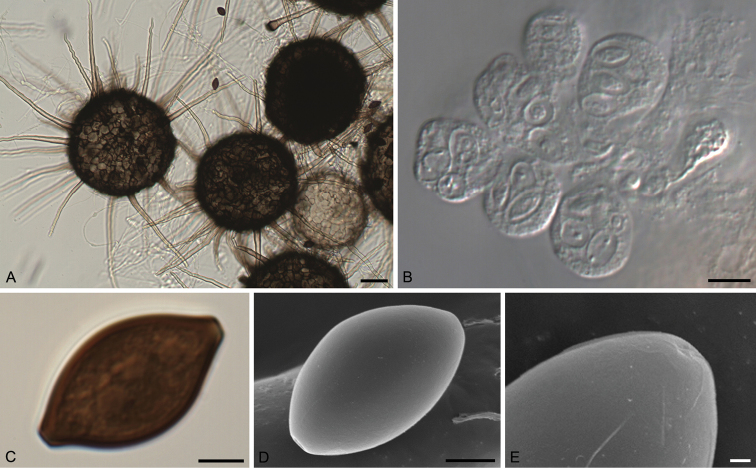
*Echinusithecacitrispora* (CBS 137837^T^). **A** Ascomata **B** Asci **C, D** Ascospores **E** Depressed germ pore. Scale bars: 50 μm (**A**); 10 μm (**B**); 5 μm (**C, D**); 1 μm (**E**).

###### Etymology.

From Latin *echinus*–, sea urchin, and from Greek –*τείχος*, wall, because of the ascomata resemblance to a sea urchin, due to the abundance of setae.

###### Notes.

This genus is characterized by dark, strongly setose, non-ostiolate ascomata. Apart from *Echinusitheca*, the other genera of the Melanosporales characterized by the production of dark semi-translucent ascomata are *Arxiomyces* and *Scopinella*, but both genera differ from *Echinusitheca* by the production of long ascomatal necks. Moreover, *Scopinella* can be easily distinguished from *Echinusitheca* by its cuboid-ellipsoidal ascospores with two prominent longitudinal germ slits, and *Arxiomyces* by its ellipsoidal ascospores that are rounded at the apex and truncated at the base, and with a broad germ pore that bears a mucilaginous and collapsing appendage.

##### Echinusitheca citrispora

Taxon classificationAnimaliaMelanosporalesCeratostomataceae

Y. Marín, Stchigel, Dania García, Guarro, A.N. Mill. & Cano sp. nov.

812085

Fig. 3

###### Type.

USA, North Carolina, Great Smoky Mountains National Park, Cataloochee Creek Campground (35.1375; -83.4915), forest soil, 15 July 2008, A.N. Miller, M. Calduch and A.M. Stchigel, holotype CBS H-21596, cultures ex-type CBS 137837 = FMR 12767.

###### Description.

Colonies on PDA attaining a diam. of 70–75 mm after 14 d at 35 °C, cottony and granulose due to the presence of a large number of ascomata, white with grey to black dots, depressed at the centre and margins fringed; reverse yellowish-white to pale yellow (4A2 to 4A3) and with olive brown (4F2) dots. Colonies on OA attaining a diam. of 50–60 mm in 14 d at 35 °C, cottony and granulose due to the presence of numerous ascomata, margins arachnoid, white to orange white (5A2) with brownish grey dots (5F2); reverse yellowish-white to golden grey (4A2 to 4C2). Minimum, maximum, and optimum temperature of growth are 20, 40 and 35 °C, respectively. *Mycelium* composed of hyaline to pale yellow, septate, branched, smooth-walled hyphae, 1–3 µm diam. *Ascomata* non-ostiolate, immersed into the mycelium, solitary or gregarious, globose, 130–280 µm diam., setose, semi-translucent, pale brown to brown, appearing black when ascospores are mature; *setae* straight, becoming sinuous toward apex, 20–200 µm long, 5–20 µm wide at base, tapering gradually to a rounded tip of 2–5 µm diam., pale brown to brown, non-septate or rarely 1-septate, thick-walled, verrucose to tuberculate, sometimes branched at apex; *ascomatal wall* membranaceous, 30–40 µm thick, composed of 5–6 layers of flattened cells of 5–30 µm diam. of *textura angularis* to *textura globulosa*. *Asci* 8-spored, globose to subglobose, 20–25 × 15–20 µm, soon evanescent, non-stipitate, without apical structures, irregularly disposed at the centrum. *Ascospores* irregularly arranged in the asci, one-celled, at first hyaline, becoming brown to dark brown when mature, smooth- and thick-walled, ellipsoidal, 20–27 × 10–15 µm, with one germ pore at each end; *germ pores* 0.75–2 µm diam., depressed. *Asexual morph* absent.

###### Etymology.

From Latin *citrum*-, lemon, and -*spora*, spore, referring to the lemon-shaped ascospores.

##### Melanospora

Taxon classificationAnimaliaMelanosporalesCeratostomataceae

Corda, Icon. fung. (Prague) 1: 24. 1837, emend.

Fig. 4

###### Type species.

*Melanosporazamiae* Corda, Icon. fung. (Prague) 1: 24. 1837. Representative strain: NBRC 7902.

###### Description.

*Ascomata* superficial to immersed, globose to subglobose, ostiolate, yellowish-orange or reddish, tomentose or glabrous, usually with a long neck composed of intermixed hypha, with a crown of rigid, hyaline, septate, smooth- and thick-walled setae; *ascomatal wall* membranaceous, translucent, of *textura angularis*. *Periphyses* present. *Paraphyses* absent. *Asci* 8-spored, clavate, rounded at apex, without apical structures, thin-walled, evanescent. *Ascospores* one-celled, at first hyaline, becoming brown to dark brown when mature, fusiform, ellipsoidal or citriform, smooth-walled, reticulate or verrucose, with a terminal apiculate or depressed germ pore at each end. *Asexual morph* phialidic, hyaline. *Bulbils* uncommon.

###### Notes.

This genus is distinguished by translucent ascomata with a neck composed of intermixed hyphae and with an apical crown of setae, smooth or ornamented ascospores with an apiculate germ pore at each end, and a phialidic asexual morph. The neck of *Melanospora* spp. is morphologically similar to those of *Syspastospora* and *Vittatispora*, which are also composed of hyphae. *Syspastospora* was introduced in 1982 by Cannon and Hawksworth to accommodate *Melanosporaparasitica*, with three additional species described later (*S.boninensis*, *S.cladoniae* and *S.tropicalis*). This genus differs from *Melanospora* in the production of cylindrical to barrel-shaped ascospores with a large, slightly sunken germ pore at both ends (ellipsoidal, citriform or fusiform, having much smaller, apiculate or depressed germ pores in *Melanospora*). *Vittatispora* can be distinguished from *Melanospora* by the production of ascospores with a thick, hyaline, longitudinal ridge and a raised rim surrounding the germ pores. Moreover, *Syspastospora* and *Vittatispora* differs from *Melanospora* in the structure of the ascomatal neck, which is composed of hyphae in a parallel arrangement in both genera (interwoven hyphae in *Melanospora*).

*Melanospora* is now restricted to species with ascoma bearing a neck composed of interwoven hyphae and mostly ending in a crown of setae. This kind of neck differentiates this genus from *Microthecium*, which has a neck composed of angular cells similar to those of the ascomatal wall and possessing a crown of setae surrounding the ostiole rather than disposed at apex of the neck. The only exception is *Melanosporamycoparasitica* that does not have this sort of neck, being short, cellular and without the crown of setae at the top of this, although this could be due to the fact that it was described and illustrated at an early stage of ascomal development. In a study on the development and cytology of *Melanosporatiffanii*, Kowalski (1965) illustrated early stages of development with the neck appearing similar to that of *M.mycoparasitica*.

Long hyphal necks are produced in *Melanosporaarenaria*, *Melanosporacaprina*, *Melanosporachionea*, *Melanosporalangenaria*, *Melanosporalongisetosa* and *Melanosporawashingtonensis*; therefore, these have been kept in the emended genus *Melanospora*, although they were not included in the phylogenetic study.

**Figure 4.**
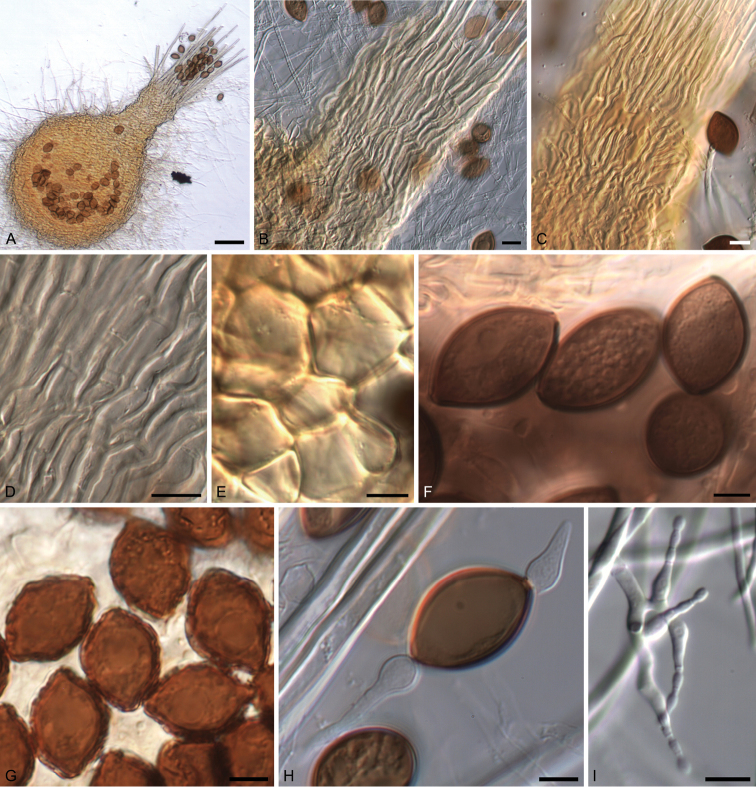
Morphological features of the genus *Melanospora*. *Melanosporadamnosa* (CBS 113681). **A** Ascoma **B** Ascomatal neck **D** Detail of hyphal neck **F** Ascospores **H** Ascospore germinating. *Melanosporazamiae* (NBRC 7902) **C** Ascomatal neck **E** Detail of ascomatal wall. *Melanosporaverrucispora* (NBRC 31375^T^) **G** Ascospores **I** Phialidic asexual morph. Scale bars: 50 μm (**A**); 10 μm (**B–E, I**); 5 μm (**F–H**).

##### Key to the species of *Melanospora*

**Table:** 

1	Ascospores with the surface ornamented	**2**
–	Ascospores smooth-walled	**4**
2	Ascospores irregularly verrucose	*** M. verrucispora ***
–	Ascospores reticulate	**3**
3	Ascospores coarsely reticulate	*** M. mycoparasitica ***
–	Ascospores slightly reticulate	*** M. tiffanii ***
4	Ascospores discoid-ellipsoidal	**5**
–	Ascospores otherwise	**7**
5	Asci 4-spored; ascospores 14–19 × 12–14 × 8–9 μm	*** M. longisetosa ***
–	Asci 8-spored; ascospores smaller	**6**
6	Neck 250–400 μm long; ascospores 7.5–16 × 6–12 × 4–7 μm	*** M. chionea ***
–	Neck 150–200(–260) μm long; ascospores 10.5–12(–13.5) × 9–10.5(–12) × 7–9 μm	*** M. washingtonensis ***
7	Ascomata usually narrower than 100 μm; ascospores citriform to rhomboidal	*** M. damnosa ***
–	Ascomata usually broader than 100 μm; ascospores ellipsoidal to citriform	**8**
8	Ascomata strongly tomentose; neck 1500–2000 μm long	*** M. caprina ***
–	Ascomata weakly or not tomentose; neck shorter than 1500 μm	**9**
9	Neck shorter than 250 μm long	*** M. zamiae ***
–	Neck longer than 800 μm long	**10**
10	Setae longer than 100 μm	*** M. arenaria ***
–	Setae up to 50 μm long	*** M. lagenaria ***

###### Melanospora arenaria

Taxon classificationAnimaliaMelanosporalesCeratostomataceae

L. Fisch. & Mont., in Montagne, Annls. Sci. Nat., Bot., sér. 4 5: 337. 1856.

####### Notes.

*Melanosporaarenaria* is characterized by ascomata with a long neck and ellipsoidal to citriform, smooth-walled ascospores. It is similar to *Melanosporacaprina*, but differs in having less tomentose ascomata with a shorter neck. Also, it is similar to *M.lagenaria*, differing only by the size of the setae at the top of the ascomatal neck. Molecular data is necessary to confirm that both species correspond to different species since the size of the setae could be influenced by the culture media on where these grew.

###### Melanospora caprina

Taxon classificationAnimaliaMelanosporalesCeratostomataceae

(Fr.) Sacc., Syll. fung. (Abellini) 2: 462. 1883.

Sphaeria caprina Fr., Fl. Danic. 11: tab. 1859, fig. 2. 1825. [Basionym]
≡ Ceratostomacaprinum (Fr.) Fr., Summa veg. Scand., Section Post. (Stockholm): 396. 1849.
≡ Cerastomacaprinum (Fr.) Quél., Mém. Soc. Émul. Montbéliard, Sér. 2 5: 522. 1875.
= Sphaeriavervecina Desm., Annls Sci. Nat., Bot., sér. 2 17: 13. 1842.
≡ Melanosporavervecina (Desm.) Fuckel, Jb. nassau. Ver. Naturk. 23-24: 126. 1870.
= Melanosporavervecina f. arundinis Sacc., Syll. fung. (Abellini) 2: 461. 1883.

####### Notes.

*Melanosporacaprina* is distinguished from the other species of the genus by its larger, white, densely tomentose ascomata with a very long neck, and ellipsoidal to citriform, smooth-walled ascospores with slightly apiculate germ pores.

###### Melanospora chionea

Taxon classificationAnimaliaMelanosporalesCeratostomataceae

(Fr.) Corda, Icon. fung. (Prague) 1: 24. 1837.

Ceratostoma chioneum Fr., Observ. mycol. (Havniae) 2: 340. 1818. [Basionym]
≡ Sphaeriachionea (Fr.) Fr., Syst. mycol. (Lundae) 2: 446. 1823.
≡ Melanosporachioneavar.chionea (Fr.) Corda, Icon. fung. (Prague) 1: 24, tab. 7, fig. 297. 1837.
= Sphaeriabiformisvar.brachystoma Pers., Syn. meth. fung. (Göttingen) 1: 60. 1801.
≡ Melanosporachioneavar.brachystoma (Pers.) Sacc., Syll. fung. (Abellini) 2: 461. 1883.
= Sphaerialeucophaea Fr., Elench. fung. (Greifswald) 2: 92. 1828.
≡ Ceratostomaleucophaeum (Fr.) Fr., Summa veg. Scand., Section Post. (Stockholm): 396. 1849.
≡ Melanosporachioneavar.leucophea (Fr.) Sacc., Syll. fung. (Abellini) 2: 461. 1883.
= Melanosporaantarctica Speg., Boln Acad. nac. Cienc. Córdoba 11: 233. 1888.

####### Notes.

This species is characterized by white, tomentose ascomata and discoid, smooth-walled ascospores with depressed germ pores.

###### Melanospora damnosa

Taxon classificationAnimaliaMelanosporalesCeratostomataceae

(Sacc.) Lindau, in Engler & Prantl, Nat. Pflanzenfam., Teil. I (Leipzig) 1: 353. 1897.

Fig. 4A, B, D, F, H

Sphaeroderma damnosum Sacc., Riv. Patol. veg. 4: 64. 1895. [Basionym]

####### Notes.

*Melanosporadamnosa* is distinguised by the production of ascomata with a short neck and citriform to rhomboidal, smooth-walled ascospores with a slightly apiculate germ pore at each end.

###### Melanospora lagenaria

Taxon classificationAnimaliaMelanosporalesCeratostomataceae

(Pers.) Fuckel, Jb. nassau. Ver. Naturk. 23-24: 126. 1870.

Sphaeria lagenaria Pers., Syn. meth. fung. (Göttingen) 1: 58. 1801. [Basionym]
≡ Ceratostomalagenaria (Pers.) Fr. [as ‘lagenarium’], Syst. veg., Edn 16: 392. 1827.
≡ Auerswaldialagenaria (Pers.) Rabenh., Hedwigia 1: 116. 1857.
≡ Cerastomalagenaria (Pers.) Quél., Mém. Soc. Émul. Montbéliard, Sér. 2 5: 522. 1875.
≡ Phaeostomalagenaria (Pers.) Munk [as ‘lagenarium’], Dansk bot. Ark. 17: 82. 1957.
= Melanosporalagenariavar.tetraspora Rehm, Hedwigia 30: 259. 1891.

####### Notes.

*Melanosporalagenaria* is similar to *M.caprina*, but the former has less tomentose ascomata with shorter necks ending in a poorly developed crown of setae. This species is also similar to *M.arenaria*. For morphological comparison see Notes of the latter species.

###### Melanospora longisetosa

Taxon classificationAnimaliaMelanosporalesCeratostomataceae

P.F. Cannon & D. Hawksw., J. Linn. Soc., Bot. 84: 130. 1982.

####### Notes.

This species is characterized by the formation of 4-spored asci and discoid, smooth-walled ascospores.

###### Melanospora mycoparasitica

Taxon classificationAnimaliaMelanosporalesCeratostomataceae

(Vujan.) Y. Marín, Stchigel, Guarro & Cano comb. nov.

812086

Sphaerodes mycoparasitica Vujan., Mycol. Res. 113: 1173. 2009. [Basionym]

####### Notes.

*Melanosporamycoparasitica* is distinguished by its fusiform, coarsely reticulate ascospores.

###### Melanospora tiffanii

Taxon classificationAnimaliaMelanosporalesCeratostomataceae

Kowalski, Mycologia 57: 279. 1965.

####### Notes.

This species is distinguished by its fusiform, slightly reticulate ascospores.

###### Melanospora verrucispora

Taxon classificationAnimaliaMelanosporalesCeratostomataceae

Takada, in Kobayasi et al., Bull. natn. Sci. Mus., Tokyo 16: 525. 1973.

Fig. 4G, I

####### Notes.

This species is characterized by irregularly verrucose ascospores.

###### Melanospora washingtonensis

Taxon classificationAnimaliaMelanosporalesCeratostomataceae

Nitzan, J.D. Rogers & D.A. Johnson, Sydowia 56: 282. 2004.

####### Notes.

This species is similar to *M.chionea*, but they differ in the length of the neck [150–200(–266) μm in *M.washingtonensis* vs. 250–400 μm in *M.chionea*] and in the size of the ascospores [10.5–12(–13.5) × 9–10.5(–12) × 7–9 μm in *M.washingtonensis* vs. 7.5–16 × 6–12 × 4–7 μm in *M.chionea*], as well as in the presence of a phialidic asexual morph in *M.washingtonensis*.

###### Melanospora zamiae

Taxon classificationAnimaliaMelanosporalesCeratostomataceae

Corda., Icon. fung. (Prague) 1: 24. 1837.

Fig. 4C, E

= Melanosporaleucotricha Corda, Icon. fung. (Prague) 1: 25. 1837.
= Melanosporacoemansii Westend., Bull. Acad. R. Sci. Belg., Cl. Sci., sér. 2 2: 579. 1857.
= Melanosporacirrhata Berk. in Cooke, Grevillea 16: 102. 1888.
= Melanosporaglobosa Berl., Malpighia 5: 409. 1891.
= Melanosporapampeana Speg., Anal. Mus. nac. Hist. nat. B. Aires 6: 287. 1898.
= Melanosporatownei Griffiths, Bull. Torrey bot. Club 26: 434. 1899.
= Melanosporarhizophila Peglion & Sacc., Annls mycol. 11: 16. 1913.
= Melanosporamattiroloana Mirande [as ‘mattiroliana’], Bull. Soc. mycol. Fr. 32: 72. 1916.
= Melanosporaschmidtii Sacc., Syll. fung. (Abellini) 24: 650. 1926.
= Melanosporaasclepiadis Zerova, J. Inst. Bot. Acad. Sci. Ukraine 12: 155. 1937.

####### Notes.

*Melanosporazamiae* is characterized by the production of ellipsoidal to citriform, smooth-walled ascospores with a depressed germ pore at each end. Doguet (1955) described the presence of bulbils; however, later studies did not mention the presence of such sort of propagules (Calviello 1973, Cannon and Hawksworth 1982), which rarely occur in the genus.

###### Doubtful species

####### Melanospora aculeata

Taxon classificationAnimaliaMelanosporalesCeratostomataceae

E.C. Hansen, Vidensk. Meddel. Dansk Naturhist. Foren. Kjøbenhavn 59: 15. 1877.

######## Notes.

Cultures of this species are not available, but it was originally described as producing small asci (18–21 × 7–8 μm) and ascospores (4–6 × 3–4 μm). This species produced ostiolate ascomata without a neck, typical of *Microthecium*; however, such small ascospores have never been seen in *Microthecium*.

####### Melanospora endobiotica

Taxon classificationAnimaliaMelanosporalesCeratostomataceae

Woron., Notul. syst. Inst. cryptog. Horti bot. petropol. 3: 31. 1924.

######## Notes.

Cultures are not available, and no illustrations were included in the protologue. It was reported as morphologically similar to *Melanosporarhizophila* [now considered a synonym of *Melanosporazamiae* (Doguet 1955)] when it was first described (Woronichin 1924).

###### Excluded species

####### Melanospora arachnophila

Taxon classificationAnimaliaMelanosporalesCeratostomataceae

Fuckel, Jb. nassau. Ver. Naturk. 23–24: 127. 1870.

######## Notes.

This species possesses cylindrical asci and hyaline ascospores, features never seen in *Melanospora.* It was previously excluded from *Melanospora* by Doguet (1955).

####### Melanospora argadis

Taxon classificationAnimaliaMelanosporalesCeratostomataceae

Czerepan., Nov. sist. Niz. Rast. 3: 177. 1966.

######## Notes.

This species shows morphological features never observed in *Melanospora*, e.g. small asci (10–14 × 5–6.5 μm) and olivaceous ascospores (5–5.5 × 3–3.5 μm). The original description is not detailed enough to ascertain its possible taxonomical placement.

####### Melanospora exsola

Taxon classificationAnimaliaMelanosporalesCeratostomataceae

Bat. & H.P. Upadhyay, Atas Inst. Micol. Univ. Recife 2: 331. 1965.

######## Notes.

This species is excluded from *Melanospora* due to its dark brown, non-translucent, setose ascomata and its small ascospores (4.5–12 × 4–7 μm), which seem to indicate a closer relationship with *Chaetomium*.

####### Melanospora gigantea

Taxon classificationAnimaliaMelanosporalesCeratostomataceae

(Massee & Crossl.) Massee & Crossl., Fungus Flora of Yorkshire (Leeds): 215. 1905.

######## Notes.

Descriptions of this species and of its basionym, *Sphaerodermagigantea*, were not found.

####### Melanospora lucifuga

Taxon classificationAnimaliaMelanosporalesCeratostomataceae

(Jungh.) Sacc., Syll. fung. (Abellini) 2: 464. 1883.

######## Notes.

Cultures are not available, and the original description does not mention asci and ascospores. Therefore, we agree with Doguet (1955) in the exclusion of this fungus from *Melanospora*.

####### Melanospora kurssanoviana

Taxon classificationAnimaliaMelanosporalesCeratostomataceae

(Beliakova) Czerepan., Notul. syst. Sect. cryptog. Inst. bot. Acad. Sci. U.S.S.R. 15: 84. 1962.

######## Notes.

In our phylogenetic study, *M.kurssanoviana* was placed in an independent lineage far from *Melanospora*. Unfortunately, the only living culture available is sterile. We did not find any distinctive morphological feature to differentiate this species from other members of the Melanosporales in the original description and in the drawing to introduce it as a new genus.

####### Melanospora macrospora

Taxon classificationAnimaliaMelanosporalesCeratostomataceae

P. Karst., Hedwigia 30: 299. 1891.

######## Notes.

Doguet (1955) excluded this species due to the production of very large cylindrical asci (480–500 × 33–36 μm) and ascospores (42–52 × 28–35 μm), morphological features not observed in any other member of the Melanosporales.

####### Melanospora octahedrica

Taxon classificationAnimaliaMelanosporalesCeratostomataceae

Pat., Cat. Rais. Pl. Cellul. Tunisie (Paris): 109. 1897.

######## Notes.

This species is transferred to *Scopinella* due to the morphology of its ascospores, i.e. octahedral ascospores with two prominent longitudinal germ slits.

####### Scopinella octahedrica

Taxon classificationAnimaliaMelanosporalesCeratostomataceae

(Pat.) Y. Marín, Stchigel, Guarro & Cano comb. nov.

812087

######## Basionym.

*Melanosporaoctahedrica* Pat., Cat. Rais. Pl. Cellul. Tunisie (Paris): 109. 1897.

####### Melanospora pascuensis

Taxon classificationAnimaliaMelanosporalesCeratostomataceae

Stchigel & Guarro, Mycol. Res. 103: 1305. 1999.

######## Notes.

This species is excluded from *Melanospora* since its neck is cellular or absent, instead it is characterized by a dark ring-like structure around the germ pores of the ascospores (Stchigel et al. 1999). This fungus could represent a new genus since such structure is unique in the Melanosporales, and these kind of structures resulted in being phylogenetically informative, as in the case of *Dactylidispora*, which is distinguished by its ascospores with a raised rim around the germ pores. The type strain of this specimen was contaminated with another fungus and it could not be included in the molecular study.

####### Melanospora setchellii

Taxon classificationAnimaliaMelanosporalesCeratostomataceae

(Harkn.) Sacc. & P. Syd., Syll. fung. (Abellini) 16: 564. 1902.

######## Notes.

This species is excluded from *Melanospora* since it produces cylindrical asci with the ascospores uniseriately disposed, a feature never observed in this genus.

####### Melanospora vitrea

Taxon classificationAnimaliaMelanosporalesCeratostomataceae

(Corda) Sacc., Syll. fung. (Abellini) 2: 463. 1883.

Sphaeronaema vitreum Corda, Icon. fung. (Prague) 1: 25. 1837. [Basionym]

######## Notes.

Doguet (1955) excluded this species due to its oblong, pale yellow ascospores.

####### Microthecium

Taxon classificationAnimaliaMelanosporalesCeratostomataceae

Corda, Icon. fung. (Prague) 5: 30, 74. 1842, emend.

Fig. 5

= Sphaerodes Clem., Gen. fung. (Minneapolis): 44, 173. 1909.
= Pteridiosperma J.C. Krug & Jeng, Mycotaxon 10: 44. 1979.
= Persiciospora P.F. Cannon & D. Hawksw., J. Linn. Soc., Bot. 84: 133. 1982.

######## Type species.

*Microtheciumzobelii* Corda, Icon. fung. (Prague) 5: 74. 1842. Representative strain: NBRC 9442.

######## Description.

*Ascomata* ostiolate or not, superficial or immersed, globose to subglobose or pyriform, yellowish-orange, orange-brown or reddish, tomentose or glabrous; *necks* short or absent, conical, composed of angular cells similar to those of the ascomatal wall, usually with a crown of hyaline, septate, smooth- and thick-walled setae around the ostiole; *ascomatal wall* membranaceous, translucent, of *textura angularis*. *Periphyses* present. *Paraphyses* absent. *Asci* 8-spored, clavate, rounded at apex, without apical structures, thin-walled, evanescent. *Ascospores* one-celled, at first hyaline, becoming brown to dark brown when mature, ellipsoidal, fusiform, navicular, citriform, plataniform or spindle-shaped, smooth, reticulate, pitted or wrinkled, with a terminal apiculate or depressed germ pore at each end. *Asexual morph* phialidic, hyaline. *Bulbils* usually produced, pale orange to reddish-orange.

**Figure 5.**
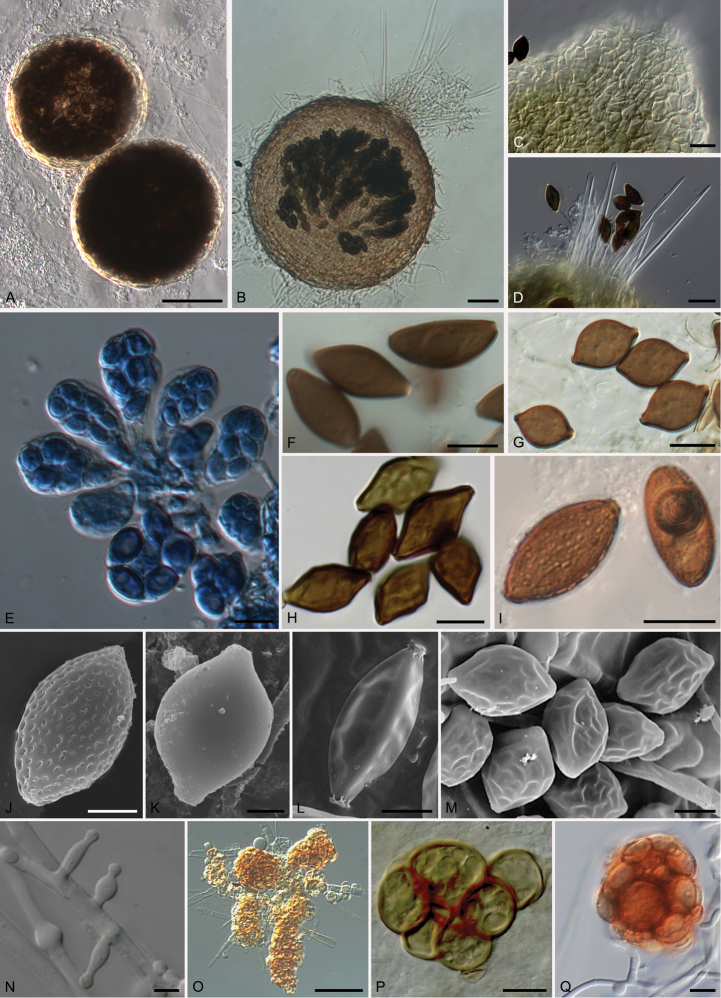
Morphological features of the genus *Microthecium*. *Microtheciumlevitum* (FMR 10098). **A** Non-ostiolate ascoma **E** Asci **G** Ascospores **K** Ascospore (SEM). *Microtheciumfayodii* (FMR 12363). **B** Ostiolate ascomata **F** Ascospores **O** Variable shaped bulbils. *Microtheciumfimicola* (FMR 5483). **C** Detail of cellular neck **M** Ascospores (SEM) **P** Bulbil. *Microtheciumquadrangulatum* (CBS 112763^T^). **D** Crown of setae around the ostiole **L** Ascospore SEM. *Microtheciumretisporum* (NBRC 8366). **H** Ascospores **N** Asexual morph. *Microtheciumjaponicum* (FMR 12371) **I** Ascospores **J** Ascospore SEM. *Microtheciumsepedonioides* (FMR 11933) **Q** Bulbil. Scale bars: 50 μm (**A, B, O**); 20 μm (**C, D**); 10 μm (**E–I, P, Q**); 5 μm (**J, L–N**); 2.5 μm (**K**).

######## Notes.

*Microthecium* has translucent ascomata of *textura angularis*, cellular necks short or absent, ascospores smooth-walled or ornamented with a depressed or apiculate germ pore at each end, often producing bulbils and a phialidic asexual morph. *Dactylidispora*, *Pustulipora* and *Pseudomicrothecium* produce ascomata similar to *Microthecium*. However, the two first genera can be distinguished by the presence of a raised rim and blistered structure surrounding the germ pores of the ascospores, respectively, while *Pseudomicrothecium* differs in the production of 2-spored asci and ascospores with indistinct germ pores.

The species *Mi.africanum*, *Mi.beatonii*, *Mi.brevirostratum*, *Mi.episphaerium*, *Mi.foveolatum*, *Mi.geoporae*, *Mi.hypomyces*, *Mi.internum*, *Mi.lenticulare*, *Mi.marchicum*, *Mi.masonii*, *Mi.micropertusum*, *Mi.moureai*, *Mi.nectrioides*, *Mi.pegleri* and *Mi.perplexum* were not included in the phylogenetic study because we could not locate any specimens since the holotypes or living cultures of most of them are not available. However, these species were transferred to *Microthecium* based on their complete and well-illustrated descriptions.

##### Key to the species of *Microthecium*

**Table:** 

1	Sexual morph absent, only producing bulbils	*** Mi. sepedonioides ***
–	Sexual morph present	**2**
2	Ascomata non-ostiolate	**3**
–	Ascomata ostiolate	**13**
3	Ascospores with an ornamented surface	**4**
–	Ascospores smooth-walled or nearly so	**8**
4	Ascospores pitted and with wing-like ridges	*** Mi. foveolatum ***
–	Ascospores coarsely reticulate	**5**
5	Asci 4-spored	**6**
–	Asci 8-spored	**7**
6	Ascospores (25–)28–34(–40) × 14–18(–20) µm	*** Mi. beatonii ***
–	Ascospores 22–28 × 12–15 × 9–11 µm	*** Mi. perplexum ***
7	Ascospores 25–34 × 12–18 µm	*** Mi. episphaerium ***
–	Ascospores 17–20 × 10–12 × 7–9 µm	*** Mi. retisporum ***
8	Ascomata smaller than 120 µm	*** Mi. tenuissimum ***
–	Ascomata longer than 120 µm	**9**
9	Ascospores shorter than 20 µm	**10**
–	Ascospores longer than 20 µm	**11**
10	Ascospores 15–19 × 11–13 × 8–9 µm, with the narrow faces coarsely reticulate and the others smooth	*** Mi. compressum ***
–	Ascospores 10–17 × 8–12 × 9–10 µm, entirely smooth-walled	*** Mi. levitum ***
11	Ascospores fusiform	*** Mi. hypomyces ***
–	Ascospores citriform	**12**
12	Ascospores 28–30 × 12–13(–15) µm	*** Mi. geoporae ***
–	Ascospores 18–25 × 8.5–12 × 6–9 µm	*** Mi. zobelii ***
13	Ascospores with wing-like appendages	**14**
–	Ascospores otherwise	**15**
14	Ascospores wrinkled, (12–)13–18 × (7–)8–10 µm	*** Mi. ciliatum ***
–	Ascospores pitted, (17–)20–22(–24) × 12–14 × 10–12 µm	*** Mi. lenticulare ***
15	Ascospores ornamentated	**16**
–	Ascospores smooth-walled	**23**
16	Ascospores punctate or punctate-reticulate	**17**
–	Ascospores reticulate or striate-reticulate	**19**
17	Ascospores punctate, ellipsoidal	*** Mi. africanum ***
–	Ascospores punctate-reticulate, ellipsoidal-fusiform	**18**
18	Ascospores delicately punctate, asexual morph and bulbils present	*** Mi. japonicum ***
–	Ascospores coarsely punctate, asexual morph and bulbils absent	*** Mi. moreaui ***
19	Ascospores striate-reticulate	**20**
–	Ascospores reticulate	**21**
20	Ascospores with inconspicuous ridges forming a very coarse reticulum, 18–22(–28) × 9.5–11(–13) × 8–9 µm	*** Mi. micropertusum ***
–	Ascospores without ridges or reticulum, 26–36 × 13–17 μm	*** Mi. masonii ***
21	Ascospores with 4–6 prominent longitudinal ribs	*** Mi. quadrangulatum ***
–	Ascospores without longitudinal ribs	**22**
22	Ascospores spindle-shaped, 19.5–22 × 8.5–11 µm	*** Mi. internum ***
–	Ascospores citriform to fusiform, 14–20 × 10–17 µm	*** Mi. fimicola ***
23	Crown of setae absent	*** Mi. nectrioides ***
–	Crown of setae present around the ostiole	**24**
24	Ascospores citriform	*** Mi. marchicum ***
–	Ascospores otherwise	**25**
25	Ascospores ellipsoid to citriform, often somewhat plataniform	**26**
–	Ascospores otherwise	**28**
26	Bulbils present	*** Mi. fallax ***
–	Bulbils absent	**27**
27	Ascospores 21–34 × 11–17 µm	*** Mi. brevirostrum ***
–	Ascospores 18–22 × 9–11 µm	*** Mi. fimbriatum ***
28	Ascospores ellipsoid to fusiform	*** Mi. fusisporum ***
–	Ascospores ellipsoid to navicular	**29**
29	Ascospores (9.5–)11–12(–13) × 4–4.5 µm	*** Mi. pegleri ***
–	Ascospores longer than 15 µm	**30**
30	Ascospores 16–24 × 8–12 µm	*** Mi. fayodii ***
–	Ascospores 25–30 × 11–15 µm	*** Mi. brevirostratum ***

###### Microthecium africanum

Taxon classificationAnimaliaMelanosporalesCeratostomataceae

(J.C. Krug) Y. Marín, Stchigel, Guarro & Cano comb. nov.

812088

Persiciospora africana J.C. Krug, Mycologia 80: 416. 1988. [Basionym]

####### Notes.

*Microtheciumafricanum* is characterized by ostiolate ascomata and punctate, ellipsoidal ascospores. Two asexual morphs with different conidia have been reported: (i), 1–4(–5)-celled, globose and smooth-walled at first but becoming cylindrical and coarsely verrucose later; (ii), 1–2-celled, large, usually cylindrical and smooth-walled (Krug 1988). However, the type strain was probably not a pure culture because the SSU and LSU sequences match with different species of *Fusarium* and the pictures of the conidia type (i) resemble the chlamydospores produced by several species of this genus.

###### Microthecium beatonii

Taxon classificationAnimaliaMelanosporalesCeratostomataceae

D. Hawksw., Trans. Mycol. Soc. Japan 18: 145. 1977.

≡ Sphaerodesbeatonii (D. Hawksw.) P.F. Cannon & D. Hawksw., Bot. J. Linn. Soc. 84: 145. 1982.

####### Notes.

This species is characterized by non-ostiolate ascomata, 4-spored asci and very coarsely reticulate, citriform ascospores. These morphological features are also observed in *Microtheciumperplexum*, but this species produces ascospores with only a third of the surface coarsely reticulate while the rest remains smooth-walled. *Microtheciumepisphaerium* and *Mi.retisporum* differ from *Mi.beatonii* in the production of 8-spored asci. Moreover, *Mi.retisporum* produces a phialidic asexual morph and bulbils, which are absent in the other mentioned species, and smaller ascospores (17–20 × 10–12 × 7–9 µm) than in *Mi.beatonii* [28–34(–40) × 14–18(–20) µm], in *Mi.episphaerium* (25–34 × 12–18 µm) and in *Mi.perplexum* (22–28 × 12–15 × 9–11 µm).

###### Microthecium brevirostratum

Taxon classificationAnimaliaMelanosporalesCeratostomataceae

(Moreau) Y. Marín, Stchigel, Guarro & Cano comb. nov.

812089

Melanospora brevirostrata Moreau, Bull. Trimest. Soc. mycol. Fr. 61: 59. 1945. [Basionym]

####### Notes.

*Microtheciumbrevirostratum* together with *Mi.fayodii* and *Mi.pegleri* produces ostiolate ascomata, smooth-walled, ellipsoidal to navicular or citriform ascospores and bulbils. *Microtheciumbrevirostratum* is easily distinguished by ascospores with apiculate germ pores and the presence of a phialidic asexual morph (ascospores show depressed germ pores and lack an asexual morph in other species). *Microtheciumfayodii* and *Mi.pegleri* differ in the size of the ascospores, *Mi.pegleri* having the smallest ascospores in *Microthecium* [(9.5–)11–12(–13) × 4–4.5 µm].

###### Microthecium brevirostrum

Taxon classificationAnimaliaMelanosporalesCeratostomataceae

(Fuckel) Y. Marín, Stchigel, Guarro & Cano comb. nov.

812090

Ceratostoma brevirostre Fuckel, Bot. Ztg. 19: 250. 1861. [Basionym]
≡ Melanosporabrevirostris (Fuckel) Höhn., Sber. Akad. Wiss. Wien, Math.-naturw. Kl., Abt. 1 123: 94. 1914.
= Ceratostomahelvellae Cooke, Grevillea 1: 175. 1873.
≡ Melanosporahelvellae (Cooke) Sacc., Syll. fung. (Abellini) 2: 462. 1883.
= Melanosporasphaerodermoides Grove, J. Bot., Lond. 23: 132. 1885.
= Melanosporasphaerodermoidesvar.sphaerodermoides Grove, J. Bot., Lond. 23: 132. 1885.
= Thielaviasoppittii Crossl., Naturalist, London: 7. 1901.
= Roselliniaaurea McAlpine, Fungus Diseases of stone-fruit trees in Australia: 102. 1902.
≡ Sphaerodermaaureum (McAlpine) Sacc. & D. Sacc., Syll. fung. (Abellini) 17: 781. 1905.
≡ Melanosporaaurea (McAlpine) Doguet, Botaniste 39: 124. 1955.
= Melanosporasphaerodermoidesvar.rubella Pidopl., Mikrobiol. Zh. 9: 61. 1948.
= Melanosporacamelina Faurel & Schotter, Revue Mycol., Paris 30: 144. 1965.
= Melanosporatulasnei Udagawa & Cain, Can. J. Bot. 47: 1932. 1970.

####### Notes.

*Microtheciumbrevirostrum*, *Mi.fallax* and *Mi.fimbriatum* produce ostiolate ascomata and ellipsoidal to citriform, often plataniform, smooth-walled ascospores with an apiculate germ pore at each end. *Microtheciumfimbriatum* is easily distinguished by its smaller (100–110 µm diam.), reddish ascomata, while *Mi.fallax* differs in the production of bulbils.

###### Microthecium ciliatum

Taxon classificationAnimaliaMelanosporalesCeratostomataceae

Udagawa & Takada, Trans. Mycol. Soc. Japan 15: 23. 1974.

≡ Pteridiospermaciliatum (Udagawa & Y. Takada) J.C. Krug & Jeng, Mycotaxon 10: 45. 1979.

####### Notes.

This species is characterized by non-ostiolate ascomata and ellipsoidal to fusiform ascospores ornamented with wing-like appendages and wrinkles, and the production of a phialidic asexual morph and bulbils. *Microtheciumlenticulare* and *Mi.foveolatum* also present ascospores with wing-like appendages, but these are pitted and not wrinkled (as in *Mi.ciliatum*), and neither species produces bulbils. *Microtheciumfoveolatum* and *Mi.ciliatum* are characterized by non-ostiolate ascomata and the production of a phialidic asexual morph, whereas *Mi.lenticulare* has ostiolate ascomata and lacks an asexual morph.

###### Microthecium compressum

Taxon classificationAnimaliaMelanosporalesCeratostomataceae

Udagawa & Cain, Can. J. Bot. 47: 1921. 1970.

≡ Sphaerodescompressa (Udagawa & Cain) P.F. Cannon & D. Hawksw., J. Linn. Soc., Bot. 84: 145. 1982.

####### Notes.

This species is distinguished by the production of non-ostiolate ascomata and citriform, bilaterally flattened ascospores, with the narrow faces coarsely reticulate and the widest faces smooth or nearly so, along with the production of a phialidic asexual morph.

###### Microthecium episphaerium

Taxon classificationAnimaliaMelanosporalesCeratostomataceae

(W. Phillips & Plowr.) Höhn., Sber. Akad. Wiss. Wien, Math.-naturw. Kl., Abt. 1 123: 98. 1914.

Melanospora episphaeria W. Phillips & Plowr., Grevillea 10: 71. 1881. [Basionym]
≡ Sphaerodermaepisphaerium (W. Phillips & Plowr.) Sacc., Syll. fung. (Abellini) 2: 460. 1883.
≡ Sphaerodesepisphaerium (W. Phillips & Plowr.) Clem. [as ‘episphaericum’], Gen. fung. (Minneapolis): 1‒227. 1909.
≡ Vittadinulaepisphaeria (W. Phillips & Plowr.) Clem. & Shear, Gen. fung., Edn 2 (Minneapolis): 281. 1931.
= Sphaerodermaepimyces Höhn., Sitzungsberichte der Kaiserlichen Akademie der Wissenschaften Math.-naturw. Klasse Abt. I 116: 103. 1907.
≡ Melanosporaepimyces (Höhn.) Doguet, Botaniste 39: 125. 1955.

####### Notes.

*Microtheciumepisphaerium* shows non-ostiolate ascomata and very coarsely reticulate, citriform ascospores. For morphological comparison see Notes of *Mi.beatonii*.

###### Microthecium fallax

Taxon classificationAnimaliaMelanosporalesCeratostomataceae

(Zukal) Y. Marín, Stchigel, Guarro & Cano comb. nov.

812772

Melanospora fallax Zukal, Sber. Akad. Wiss. Wien, Math.-naturw. Kl., Abt. 1 98: 547. 1889. [Basionym]
= Melanosporaanomala Hotson, Proc. Amer. Acad. Arts & Sci 48.: 257. 1912.
= Melanospora cervicula Hotson, Proc. Amer. Acad. Arts & Sci. 48: 254. 1912.
= Melanosporapapillata Hotson, Proc. Amer. Acad. Arts & Sci 48.: 251. 1912.
= Melanosporaphaseoli Roll-Hansen, Blyttia 6: 73. 1948.

####### Notes.

This species is characterized by ostiolate ascomata, ellipsoidal to citriform, often plataniform, smooth-walled ascospores, and production of bulbils. For morphological comparison see Notes of *Mi.brevirostrum*.

###### Microthecium fayodii

Taxon classificationAnimaliaMelanosporalesCeratostomataceae

(Vuill.) Y. Marín, Stchigel, Guarro & Cano comb. nov.

812091

Fig. 5B, F, O

Melanospora fayodii Vuill. [as ‘fayodi’], Bull. Séanc. Soc. Sci. Nancy, Sér. 2 8: 33. 1887. [Basionym]

####### Notes.

This species is characterized by ostiolate ascomata, ellipsoidal to navicular or citriform, smooth-walled ascospores, and production of bulbils. For morphological comparison see Notes of *Mi.brevirostratum*.

###### Microthecium fimbriatum

Taxon classificationAnimaliaMelanosporalesCeratostomataceae

(Rostr.) Y. Marín, Stchigel, Guarro & Cano comb. nov.

812092

Sphaeroderma fimbriatum Rostr., Oest. Grönl. Svampe: 25. 1894. [Basionym]
≡ Melanosporafimbriata (Rostr.) Petch, Trans. Br. mycol. Soc. 21: 253. 1938.

####### Notes.

*Microtheciumfimbriatum* produces ostiolate ascomata, and citriform to plataniform, smooth-walled ascospores with a strongly apiculate and tuberculate germ pore at each end. Although the ascomata was described as small and reddish in the protologue, the strain included in this study (NBRC 8523) shows larger (250–380 µm diam.), orange-brown ascomata. Moreover, our isolate produces bulbils. For morphological comparison see Notes of *Mi.brevirostrum*.

###### Microthecium fimicola

Taxon classificationAnimaliaMelanosporalesCeratostomataceae

(E.C. Hansen) Y. Marín, Stchigel, Guarro & Cano comb. nov.

812093

Fig. 5C, M, P

Melanospora fimicola E.C. Hansen, Vidensk. Meddel. Dansk Naturhist. Foren. Kjøbenhavn 59: 15. 1876. [Basionym]
≡ Sphaerodermafimicola (E.C. Hansen) Sacc., Syll. fung. (Abellini) 2: 460. 1883.
≡ Sphaerodesfimicola (E.C. Hansen) P.F. Cannon & D. Hawksw., J. Linn. Soc., Bot. 84: 146. 1982.
= Melanosporaornata Zukal, Verh. zool.-bot. Ges. Wien 35: 340. 1886.
≡ Sphaerodesornata (Zukal) Arx, Gen. Fungi Sporul. Cult., Edn 3 (Vaduz): 156. 1981.
= Sphaerodermahulseboschii Oudem., Contrib. Flora Mycol. d. Pays-Bas 11: 23. 1886.
≡ Melanosporahulseboschii (Oudem.) Doguet, Botaniste 39: 121. 1955.
= Melanosporaaffine Sacc. & Flageolet, Bull. Soc. Mycol. Fr. 12: 67. 1896.
= Melanosporamanginii Vincens [as ‘mangini’], Bull. Soc. Mycol. Fr. 33: 69. 1917.
≡ Sphaerodesmanginii (Vincens) Arx, Gen. Fungi Sporul. Cult., Edn 3 (Vaduz): 156. 1981.

####### Notes.

*Microtheciumfimicola* is characterized by ostiolate ascomata and coarsely reticulate ascospores with strongly apiculate germ pores at both ends. The other species with ostiolate ascomata and reticulate ascospores are *Mi.internum* and *Mi.quadrangulatum*. The main differences among them are the shape and size of the ascospores, being citriform in *Mi.fimicola*, spindle-shaped in *Mi.internum* and fusiform in *Mi.quadrangulatum*. The production of bulbils has only been observed in our fresh isolates of *Mi.fimicola*, although this was not previously reported.

###### Microthecium foveolatum

Taxon classificationAnimaliaMelanosporalesCeratostomataceae

Udagawa & Y. Horie, in Hawksworth & Udagawa, Trans. Mycol. Soc. Japan 18: 149. 1977.

≡ Pteridiospermafoveolatum (Udagawa & Y. Horie) J.C. Krug & Jeng, Mycotaxon 10: 45. 1979.

####### Notes.

This species is easily distinguished by its non-ostiolate ascomata, ellipsoidal to fusiform ascospores ornamented with small pores and thick wing-like ridges usually longitudinal but often oblique, and production of phialidic asexual morph. For morphological comparison see Notes of *Mi.ciliatum*.

###### Microthecium fusisporum

Taxon classificationAnimaliaMelanosporalesCeratostomataceae

(Petch) Y. Marín, Stchigel, Guarro & Cano comb. nov.

812094

Sphaeroderma fusisporum Petch, Naturalist, London: 58. 1936. [Basionym]
≡ Melanosporafusispora (Petch) Doguet, Botaniste 39: 215. 1955.
= Melanosporafusisporavar.fusispora (Petch) Doguet, Botaniste 39: 215. 1955.
= Melanosporafusisporavar.parvispora Matsush., Matsush. Mycol. Mem. 8: 24. 1995.

####### Notes.

*Microtheciumfusisporum* is related to *Mi.nectrioides*, both possessing ostiolate ascomata and smooth-walled ascospores. However, *Mi.nectrioides* can be distinguished by the absence of the crown of setae around the ostiole and its citriform ascospores, being fusiform in *Mi.fusisporum*.

###### Microthecium geoporae

Taxon classificationAnimaliaMelanosporalesCeratostomataceae

(W. Oberm.) Höhn., Sber. Akad. Wiss. Wien, Math.-naturw. Kl., Abt. 1 123: 98. 1914.

Guttularia geoporae W. Oberm., Mykol. Zentbl. 3: 9. 1913. [Basionym]

####### Notes.

This species produces non-ostiolate ascomata and citriform, smooth-walled ascospores. Other species previously placed in *Melanospora* characterized by the production of non-ostiolate ascomata and smooth-walled ascospores are *Mi.hypomyces*, *Mi.levitum* and *Mi.zobelii*. *Microtheciumhypomyces* is distinguished by its fusiform ascospores (citriform in the other species), and *Mi.levitum* by the presence of bulbils and a phialidic asexual morph. *Microtheciumgeoporae* and *Mi.zobelii* are distinguished by the size of their ascospores [28–30 × 12–13(–15) µm in *Mi.geoporae* and 18–25 × 8.5–12 × 6–9 µm in *Mi.zobelii*]. *Microtheciumtenuissimum* shows similar morphological features to these species but its ascospores are finely reticulate under SEM and its ascomata are smaller (less than 120 µm) than in the other species.

###### Microthecium hypomyces

Taxon classificationAnimaliaMelanosporalesCeratostomataceae

(Höhn.) Höhn., Sber. Akad. Wiss. Wien, Math.-naturw. Kl., Abt. 1 123: 50. 1914.

Sphaeroderma hypomyces Höhn., Sber. Akad. Wiss. Wien, Math.-naturw. Kl., Abt. 1 116: 102. 1907. [Basionym]
≡ Melanosporahypomyces (Höhn.) Doguet, Botaniste 39: 215. 1955.

####### Notes.

This species is characterized by non-ostiolate ascomata and fusiform, smooth-walled ascospores. For morphological comparison see Notes of *Mi.geoporae*.

###### Microthecium internum

Taxon classificationAnimaliaMelanosporalesCeratostomataceae

(Tehon & G.L. Stout) Y. Marín, Stchigel, Guarro & Cano comb. nov.

812095

Melanospora interna Tehon & G.L. Stout, Mycologia 21: 181. 1929. [Basionym]

####### Notes.

This species produces ostiolate ascomata and spindle-shaped ascospores with a coarse and irregular reticulum. For morphological comparison see Notes of *Mi.fimicola*.

###### Microthecium japonicum

Taxon classificationAnimaliaMelanosporalesCeratostomataceae

(Y. Horie, Udagawa & P.F. Cannon) Y. Marín, Stchigel, Guarro & Cano comb. nov.

812096

Fig. 5I, J

Persiciospora japonica Y. Horie, Udagawa & P.F. Cannon, Mycotaxon 25: 233. 1986. [Basionym]

####### Notes.

*Microtheciumjaponicum* is characterized by ostiolate ascomata and ellipsoidal to fusiform, punctate-reticulate ascospores, similar to *Mi.moureai*. However, *Mi.japonicum* produces a phialidic asexual morph and bulbils (absent in *Mi.moureai*) and delicately reticulate ascospores (coarsely reticulate in *Mi.moureai*).

###### Microthecium lenticulare

Taxon classificationAnimaliaMelanosporalesCeratostomataceae

(Udagawa & T. Muroi) Y. Marín, Stchigel, Guarro & Cano comb. nov.

812097

Pteridiosperma lenticulare Udagawa & T. Muroi [as ‘lenticularis’], Trans. Mycol. Soc. Japan 22: 20. 1981. [Basionym]

####### Notes.

*Microtheciumlenticulare* produces ostiolate ascomata and pitted-walled ascospores with wing-like appendages. For morphological comparison see Notes of *Mi.ciliatum*.

###### Microthecium levitum

Taxon classificationAnimaliaMelanosporalesCeratostomataceae

Udagawa & Cain, Can. J. Bot. 47: 1917. 1970.

Fig. 5A, E, G, K

≡ Sphaerodeslevita (Udagawa & Cain) D. García, Stchigel & Guarro, Stud. Mycol. 50: 67. 2004.

####### Notes.

This species is characterized by non-ostiolate ascomata, citrifrom and smooth-walled ascospores with umbonate and tuberculate germ pores, presence of bulbils and phialidic asexual morph. For morphological comparison see Notes of *Mi.geoporae*.

###### Microthecium marchicum

Taxon classificationAnimaliaMelanosporalesCeratostomataceae

(Lindau) Y. Marín, Stchigel, Guarro & Cano comb. nov.

812099

Chaetomium marchicum Lindau, Hedwigia 35: 56. 1896. [Basionym]
≡ Sphaerodermamarchicum (Lindau) Sacc. & P. Syd., Syll. fung. (Abellini) 14: 627. 1899.

####### Notes.

*Microtheciummarchicum* is characterized by its ostiolate ascomata and citrifrom, smooth-walled ascospores. Its ascospores are similar to those of *Mi.geoporae*, *Mi.hypomyces*, *Mi.levitum* and *Mi.zobelii*, but all of them produce non-ostiolate ascomata.

###### Microthecium masonii

Taxon classificationAnimaliaMelanosporalesCeratostomataceae

(Kirschst.) Y. Marín, Stchigel, Guarro & Cano comb. nov.

812100

Ceratostoma masonii Kirschst., Trans. Br. mycol. Soc. 18: 306. 1934. [Basionym]
≡ Persiciosporamasonii (Kirschst.) P.F. Cannon & D. Hawksw., J. Linn. Soc., Bot. 84: 135. 1982.

####### Notes.

*Microtheciummasonii* is characterized by ostiolate ascomata and ellipsoidal to fusiform, faintly striate-reticulate ascospores. The same type of ascospore ornamentation is also observed in *Mi.micropertusum*, but this latter species is easily distinguished by the presence of inconspicuous ridges forming a very coarse reticulum and a phialidic asexual morph.

###### Microthecium micropertusum

Taxon classificationAnimaliaMelanosporalesCeratostomataceae

(Y. Horie, Udagawa & P.F. Cannon) Y. Marín, Stchigel, Guarro & Cano comb. nov.

812101

Sphaerodes micropertusa Y. Horie, Udagawa & P.F. Cannon, Mycotaxon 25: 236. 1986. [Basionym]

####### Notes.

*Microtheciummicropertusum* is distinguished by its ostiolate ascomata, fusiform to citriform or nearly rhombic in outline ascospores with inconspicuous ridges forming a coarse reticulum, and presence of phialidic asexual morph. For morphological comparison see Notes of *Mi.masonii*.

###### Microthecium moreaui

Taxon classificationAnimaliaMelanosporalesCeratostomataceae

(P.F. Cannon & D. Hawksw.) Y. Marín, Stchigel, Guarro & Cano comb. nov.

812102

Persiciospora moreaui P.F. Cannon & D. Hawksw., J. Linn. Soc., Bot. 84: 134. 1982. [Basionym]

####### Notes.

*Microtheciummoreaui* is characterized by its ostiolate ascomata, ellipsoidal and pitted-walled ascospores, and production of bulbils. For morphological comparison see Notes of *Mi.japonicum*.

###### Microthecium nectrioides

Taxon classificationAnimaliaMelanosporalesCeratostomataceae

(Marchal) Y. Marín, Stchigel, Guarro & Cano comb. nov.

812103

Sphaeroderma nectrioides Marchal, Bull. Soc. R. Bot. Belg. 23: 25. 1884. [Basionym]
≡ Melanosporanectrioides (Marchal) Doguet, Botaniste 39: 121. 1955.
= Melanosporaasparagi G. Arnaud, Ann. Serv. Epiph. 2: 273. 1915.

####### Notes.

This species produces ostiolate ascomata and citriform, smooth-walled ascospores. For morphological comparison see Notes of *Mi.fusisporum*.

###### Microthecium pegleri

Taxon classificationAnimaliaMelanosporalesCeratostomataceae

(D. Hawksw. & A. Henrici) Y. Marín, Stchigel, Guarro & Cano comb. nov.

812104

Melanospora pegleri D. Hawksw. & A. Henrici, Kew Bull. 54: 795. 1999. [Basionym]

####### Notes.

*Microtheciumpegleri* is characterized by ostiolate ascomata, ellipsoidal to plano-convex, smooth-walled ascospores and presence of bulbils. For morphological comparison see Notes of *Mi.brevirostratum*.

###### Microthecium perplexum

Taxon classificationAnimaliaMelanosporalesCeratostomataceae

D. Hawksw., Trans. Mycol. Soc. Japan 18: 151. 1977.

≡ Sphaerodesperplexa (D. Hawksw.) P.F. Cannon & D. Hawksw., Bot. J. Linn. Soc. 84: 148. 1982.

####### Notes.

This species produces non-ostiolate ascomata, 4-spored asci and citrifrom ascospores usually with smooth walls, but one third of these are coarsely reticulated. For morphological comparison see Notes of *Mi.beatonii*.

###### Microthecium quadrangulatum

Taxon classificationAnimaliaMelanosporalesCeratostomataceae

(D. García, Stchigel & Guarro) Y. Marín, Stchigel, Guarro & Cano comb. nov.

812105

Fig. 5D, L

Sphaerodes quadrangularis D. García, Stchigel & Guarro, Stud. Mycol. 50: 64. 2004. [Basionym]

####### Notes.

*Microtheciumquadrangulatum* is characterized by ostiolate ascomata and fusiform, reticulate ascospores with strongly apiculate germ pores. For morphological comparison see Notes of *Mi.fimicola*.

###### Microthecium retisporum

Taxon classificationAnimaliaMelanosporalesCeratostomataceae

Udagawa & Cain, Can. J. Bot. 47: 1926. 1970.

Fig. 5H, N

≡ Sphaerodesretispora (Udagawa & Cain) P.F. Cannon & D. Hawksw., J. Linn. Soc., Bot. 84: 149. 1982.
= Microtheciumretisporumvar.inferius Udagawa & Cain [as ‘inferior’], Can. J. Bot. 47: 1928. 1970.
≡ Sphaerodesretisporavar.inferior (Udagawa & Cain) P.F. Cannon & D. Hawksw., J. Linn. Soc., Bot. 84: 149. 1982.
≡ Sphaerodesinferior (Udagawa & Cain) D.W. Li & N.P. Schultes, in Schultes, Murtishi & Li, Fungal Biology 121: 901. 2017.
= Microtheciumretisporumvar.retisporum Udagawa & Cain, Can. J. Bot. 47: 1926. 1970.
≡ Sphaerodesretisporavar.retispora (Udagawa & Cain) P.F. Cannon & D. Hawksw., J. Linn. Soc., Bot. 84: 149. 1982.

####### Notes.

This species is characterized by non-ostiolate ascomata, reticulate citriform ascospores with apiculate germ pores, a phialidic asexual morph and presence of bulbils. For morphological comparison see Notes of *Mi.beatonii*.

###### Microthecium sepedonioides

Taxon classificationAnimaliaMelanosporalesCeratostomataceae

(Preuss) Y. Marín, Stchigel, Guarro & Cano comb. nov.

812106

Fig. 5Q

Papulaspora sepedonioides Preuss, Linnaea 24: 112. 1851. [Basionym]

####### Notes.

*Microtheciumsepedonioides* only produces bulbils and the sexual morph has never been observed.

###### Microthecium tenuissimum

Taxon classificationAnimaliaMelanosporalesCeratostomataceae

(D. García, Stchigel & Guarro) Y. Marín, Stchigel, Guarro & Cano comb. nov.

812107

Sphaerodes tenuissima D. García, Stchigel & Guarro, Stud. Mycol. 50: 65. 2004. [Basionym]

####### Notes.

This species is characterized by non-ostiolate ascomata and citriform, ellipsoidal in lateral view, finely reticulate ascospores with strongly apiculate germ pores. For morphological comparison see Notes of *Mi.geoporae*.

###### Microthecium zobelii

Taxon classificationAnimaliaMelanosporalesCeratostomataceae

Corda, Icon. fung. (Prague) 5: 74. 1842.

≡ Sphaeriazobelii (Corda) Tul. & C. Tul., Fungi hypog.: 186. 1851.
≡ Ceratostomazobelii (Corda) Berk., Journal of the Royal Horticultural Society 4: 402. 1860.
≡ Melanosporazobelii (Corda) Fuckel, Jb. nassau. Ver. Naturk. 23-24: 127. 1870.
= Melanosporazobeliivar.zobelii (Corda) Fuckel, Jb. nassau. Ver. Naturk. 23-24: 127. 1870.
= Melanosporacoprophila Zukal, Sber. Akad. Wiss. Wien, Math.-naturw. Kl., Abt. 1 98: 544. 1889.
= Melanosporamarchicum Lindau, Hedwigia 35: 56. 1896.
= Melanosporazobeliivar.minor Pidopl., Mikrobiol. Zh. 9(2-3): 60. 1948.

####### Notes.

*Microtheciumzobelii* produces non-ostiolate ascomata, and citriform, smooth-walled ascospores with slightly apiculate germ pores. For morphological comparison see Notes of *Mi.geoporae*.

###### Doubtful species

####### Microthecium ryvardenianum

Taxon classificationAnimaliaMelanosporalesCeratostomataceae

Aramb. & Gamundí, Agarica 6: 124. 1985.

######## Notes.

This species is considered as doubtful because it presents morphological features atypical of *Microthecium* (e.g. allantoid ascospores when immature becoming striate when mature).

####### Pseudomicrothecium

Taxon classificationAnimaliaMelanosporalesCeratostomataceae

Y. Marín, Stchigel, Guarro, Cano, gen. nov .

812108

######## Type species.

*Pseudomicrotheciumsubterraneum* (L. Fan, C.L. Hou, P.F. Cannon & Yong Li) Y. Marín, Stchigel, Guarro & Cano. Holotype and ex-isotype strain: BJTC FAN1001, K[M] 172128.

######## Description.

*Ascomata* non-ostiolate, globose, translucent, pale brown to brown, appearing dark brown when the ascospores are mature, glabrous or setose; *ascomatal wall* membranaceous, of *textura angularis*. *Asci* 2-spored, clavate, short-stipitate, without apical structures, evanescent. *Ascospores* one-celled, at first hyaline, becoming dark brown to blackish when mature, ellipsoidal to citriform, umbonate and truncate at both ends, with a terminal indistinct germ pore at each end. *Asexual morph* absent.

######## Etymology.

The name refers to the morphological resemblance to *Microthecium*.

######## Notes.

The new genus *Pseudomicrothecium* is proposed here to accommodate *Melanosporasubterranea* because it constitutes a separate lineage in our phylogenetic study. This genus is characterized by its non-ostiolate ascomata, similar to those of *Microthecium*, 2-spored asci and smooth-walled ascospores with an indistinct germ pore at each end. Asci containing two ascospores have only been observed in some species of *Scopinella* (i.e. *Scopinellagallicola* and *S.sphaerophila*). However, *Scopinella* can be easily distinguished from *Pseudomicrothecium* by the production of ostiolate ascomata with long necks and cuboid-ellipsoidal ascospores with two prominent longitudinal germ slits.

####### Pseudomicrothecium subterraneum

Taxon classificationAnimaliaMelanosporalesCeratostomataceae

(L. Fan, C.L. Hou, P.F. Cannon & Yong Li) Y. Marín, Stchigel, Guarro & Cano comb. nov.

812109

######## Basionym.

*Melanosporasubterranea* L. Fan, C.L. Hou, P.F. Cannon & Yong Li, Mycologia 104: 1434. 2012.

## Discussion

We have revised the taxonomy of relevant members of the family Ceratostomataceae based on the analyses of the SSU, LSU, ITS, *act* and *tef1* nucleotide sequences. This study strongly supported the order Melanosporales proposed by Zhang and Blackwell in 2007 (Hibbett et al. 2007). The phylogenetic inference showed seven lineages corresponding to the genera *Dactylidispora*, *Echinusitheca*, *Melanospora*, *Microthecium*, *Pseudomicrothecium* and *Vittatispora*, and to *Melanosporakurssanoviana*. Our results agree with previous studies (Zhang and Blackwell 2002, Fan et al. 2012) which already suggested and demonstrated that the ornamentation of the ascospores under SEM, a feature traditionally used to delimit most of the genera in the Melanosporales, is not useful for estimating phylogenetic relationships among these fungal taxa. Similarly, the morphology of the ascospores is of weak taxonomic value and a poor predictor for the generic delimitation of members of the family Sordariaceae, resulting in the synonymy of two relevant genera, i.e. *Gelasinospora* and *Neurospora* (Dettman et al. 2001, García et al. 2004, Nygren et al. 2011). In our study, two of the largest genera of the Melanosporales, *Melanospora* and *Microthecium*, grouped species with both smooth and ornamented ascospore walls. By contrast, a phylogenetic study of the Lasiosphaeriaceae (Miller and Huhndorf 2005) revealed that the morphology of the ascomatal wall was more phylogenetically informative than that of the ascospores, with several new genera proposed (i.e. *Immersiella*) or emended (i.e. *Lasiosphaeria*, *Lasiosphaeris* and *Schizothecium*) (Miller and Huhndorf 2004, Cai et al. 2005). Here, the erection of the new genus *Echinusitheca* is a clear example of the relevance of the ascomatal morphology in the taxonomy of these fungi, and in fact this taxon together with *Arxiomyces* and *Scopinella* are the only genera in the Melanosporales that show dark semi-transluscent ascomata. In this context, although *Echinusitheca* has ascospores similar to those of *Melanospora* and *Microthecium*, this genus constitutes one of the lineages phylogenetically most distant within this order.

Another lineage considerably distant from the other members of the Melanosporales is constituted by the clade represented only by the species *Melanosporakurssanoviana*, suggesting that this fungus could represent a new genus. However, this new taxon is at this moment not proposed because its colonies, in spite of attempts to induce sporulation, remain sterile and a detailed morphological study was not possible. The infertility of the cultures is probably due to the fact that an important part of the members of this fungal group show a peculiar habitat developing a certain degree of mycoparasitism and requiring the presence of the host to complete the biologic cycle and develope reproductive structures. The mycoparasitism of *Melanospora*, *Syspastospora* and the species previously placed in *Persiciospora* and *Sphaerodes* has already been demonstrated by numerous authors (Doguet 1955, Calviello 1973, Jordan and Barnett 1978, Harveson and Kimbrough 2000, 2001), and this ability has been exploited in the biocontrol of phytopathogenic fungi (Vujanovic and Goh 2009, Goh and Vujanovic 2010, Kim and Vujanovic 2016, 2017).

The genus *Sphaeronaemella*, which is characterized by pale and translucent ascomata, was thought to be related to *Melanospora* (Cannon and Hawksworth 1982). However, we do not agree with this relationship because it differs from the Melanosporales in the production of hyaline ascospores, as opposed to the pigmented ones in the members of that order. By contrast, our results correlate with those of other authors that demonstrated a closer phylogenetic relationship of this genus with the members of the order Microascales (Spatafora and Blackwell 1994b, Hausner and Reid 2004). In fact, our SSU tree seems to indicate that *Sphaeronaemella* could represent a new family of the Microascales; however, further studies including more taxa and additional genes are needed to more accurately confirm its taxonomic status.

The placement of our isolate of *Persiciosporajaponicum* in the *Microthecium* clade once more demonstrated that the ornamentation of the ascospores, which is pitted in *Persiciospora* spp., is of poor taxonomic value, and consequently all the species of *Persiciospora* should be transferred to *Microthecium*. As it was above mentioned, the species of this latter genus show a typical cellular ascomatal neck which is also present in *Persiciospora* and constitutes a common feature in both genera. Surprisingly, in some previous phylogenetic studies, the species of *Persiciospora* were placed in the Hypocreales, closely related to *Nectria* (Zhang and Blackwell 2002, Maharachchikumbura et al. 2015, Schultes et al. 2017). However, this could be probably explained by a possible contamination of the cultures of *Persiciospora* spp. with an hypocrealean host (Fan et al. 2012). The same situation may have occurred with the cultures of *Scopinella* and *Syspastospora*, which led to a possible erroneous classification of both taxa in the Hypocreales (Zhang and Blackwell 2002, Chaudhary et al. 2006, Fan et al. 2012, Maharachchikumbura et al. 2015, Schultes et al. 2017).

*Pteridiospermaciliatum*, a member of the Melanosporales with ascospores ornamented with longitudinal wing-like ridges that anastomose each other to form a well defined reticulum (a distinctive feature of *Pteridiosperma*), was also found in the *Microthecium* clade, proving once again that the ascospore ornamentation is not phylogenetically informative. Consequently, we have synonymyzed the genus *Pteridiosperma* with *Microthecium* since *Pteridiosperma* spp. show non-ostiolate ascomata, or if ostiolate, they show a short neck composed of angular cells, which are typical morphological characteristics of *Microthecium*.

Another genus that our results demonstrated should be synonymized and included in *Microthecium* is *Sphaerodes* because its type species, *S.episphaerium*, shows morphological features (non-ostiolate ascomata) that fit with the current circumscription of that emended genus. Most of the species of *Sphaerodes*, with the exception of *S.ellipsospora* and *S.singaporensis*, which are now located in the new genus *Dactylidispora*, and *S.mycoparasitica*, which is now placed in *Melanospora*, are also transferred to *Microthecium* since these produce non-ostiolate or ostiolate ascomata without a neck, or less frequently with a short neck composed of angular cells similar to the ascomatal ones. Another relevant feature of the genus *Microthecium* is the production of bulbils. These propagules are typical of *Papulaspora*, an anamorphic genus that encompasses more than 40 species. Although it was initially accepted as a genus without a sexual morph (Hotson 1912), its link with species of *Melanospora* and *Chaetomium* has been reported (Roll-Hansen 1948, Zhang et al. 2004). In our phylogenetic study *Papulasporasepedonioides*, the type species of the genus, was nested in the *Microthecium* clade, and therefore transferred to this genus. The relationship of this species with the Melanosporales had already previously been demonstrated by Davey et al. (2008) and Li et al. (2016). However, it has been demonstrated that *Papulaspora* is a polyphyletic genus, and other species of the genus have been reported as belonging to the classes Leotiomycetes and Sordariomycetes (Ascomycota). Therefore, the other species of *Papulaspora* not linked to the species of *Microthecium* should be transferred to other taxonomic groups. The relationship of some species of *Papulaspora* with the Melanosporales is also suggested by the production of similar phialidic synanamorphs (Van Beyma 1931, Hotson 1942).

The most recent new combination performed in *Sphaerodes*, *S.inferior*, was done to accommodate S.retisporavar.inferior since it was not clustering with S.retisporavar.retispora (Schultes et al. 2017). However, we suspected that the sequences of S.retisporavar.retispora deposited in GenBank were contaminated with the hypocrealean host. In order to corroborate it, we studied such sequenced strain demonstrating that it was effectively contaminated. Therefore, *S.inferior* is here considered a synonym of *Mi.retisporum* since the morphological differences are insufficient to recognize this variety as a different species.

There are important morphological differences among the strains of *Microthecium* that suggest the presence of several additional cryptic species in the genus; however, our phylogenetic study, in spite of having used five loci, was not able to resolve the boundaries among them.

## Supplementary Material

XML Treatment for Dactylidispora

XML Treatment for Dactylidispora collipora

XML Treatment for Dactylidispora ellipsospora

XML Treatment for Dactylidispora singaporensis

XML Treatment for Echinusitheca

XML Treatment for Echinusitheca citrispora

XML Treatment for Melanospora

XML Treatment for Melanospora arenaria

XML Treatment for Melanospora caprina

XML Treatment for Melanospora chionea

XML Treatment for Melanospora damnosa

XML Treatment for Melanospora lagenaria

XML Treatment for Melanospora longisetosa

XML Treatment for Melanospora mycoparasitica

XML Treatment for Melanospora tiffanii

XML Treatment for Melanospora verrucispora

XML Treatment for Melanospora washingtonensis

XML Treatment for Melanospora zamiae

XML Treatment for Melanospora aculeata

XML Treatment for Melanospora endobiotica

XML Treatment for Melanospora arachnophila

XML Treatment for Melanospora argadis

XML Treatment for Melanospora exsola

XML Treatment for Melanospora gigantea

XML Treatment for Melanospora lucifuga

XML Treatment for Melanospora kurssanoviana

XML Treatment for Melanospora macrospora

XML Treatment for Melanospora octahedrica

XML Treatment for Scopinella octahedrica

XML Treatment for Melanospora pascuensis

XML Treatment for Melanospora setchellii

XML Treatment for Melanospora vitrea

XML Treatment for Microthecium

XML Treatment for Microthecium africanum

XML Treatment for Microthecium beatonii

XML Treatment for Microthecium brevirostratum

XML Treatment for Microthecium brevirostrum

XML Treatment for Microthecium ciliatum

XML Treatment for Microthecium compressum

XML Treatment for Microthecium episphaerium

XML Treatment for Microthecium fallax

XML Treatment for Microthecium fayodii

XML Treatment for Microthecium fimbriatum

XML Treatment for Microthecium fimicola

XML Treatment for Microthecium foveolatum

XML Treatment for Microthecium fusisporum

XML Treatment for Microthecium geoporae

XML Treatment for Microthecium hypomyces

XML Treatment for Microthecium internum

XML Treatment for Microthecium japonicum

XML Treatment for Microthecium lenticulare

XML Treatment for Microthecium levitum

XML Treatment for Microthecium marchicum

XML Treatment for Microthecium masonii

XML Treatment for Microthecium micropertusum

XML Treatment for Microthecium moreaui

XML Treatment for Microthecium nectrioides

XML Treatment for Microthecium pegleri

XML Treatment for Microthecium perplexum

XML Treatment for Microthecium quadrangulatum

XML Treatment for Microthecium retisporum

XML Treatment for Microthecium sepedonioides

XML Treatment for Microthecium tenuissimum

XML Treatment for Microthecium zobelii

XML Treatment for Microthecium ryvardenianum

XML Treatment for Pseudomicrothecium

XML Treatment for Pseudomicrothecium subterraneum

## References

[B1] Alexopoulos CJ (1962) Introductory mycology. 2^nd^ edn. John Wiley, New York, 613 pp.

[B2] Bessey EA (1950) Morphology and Taxonomy of Fungi. Blakiston, Philadelphia, Toronto, 791 pp 10.5962/bhl.title.5663

[B3] Cai L Jeewon R Hyde KD (2005) Phylogenetic evaluation and taxonomic revision of *Schizothecium* based on ribosomal DNA and protein coding genes. Fungal Diversity 19: 1–21.

[B4] Calviello BO (1973) Contribución al estudio de ascomycetes argentinos. I. Comunicaciones del Museo Argentino de Ciencias Naturales ‘Bernardino Rivadavia’. Botánica 2: 31–39.

[B5] Cannon PF (1982) *Pustulipora*, a new genus of the Melanosporaceae. Mycotaxon 15: 523–528.

[B6] Cannon PF Hawksworth DL (1982) A re-evaluation of *Melanospora* Corda and similar pyrenomycetes, with a revision of the British species. The Journal of the Linnean Society, Botany 84: 115–160. 10.1111/j.1095-8339.1982.tb00363.x

[B7] Cannon PF Hawksworth DL (1983) *Arxiomyces*, a new name for *Phaeostoma* von Arx & E. Müller. Transactions of the British Mycological Society 8(3): 644–645. 10.1016/S0007-1536(83)80143-0

[B8] Cano J Guarro J Gené J (2004) Molecular and morphological identification of *Colletotrichum* species of clinical interest. Journal of Clinical Microbiology 42(6): 2450–2454. 10.1128/JCM.42.6.2450-2454.2004 15184418 PMC427825

[B9] Chaudhary P Campbell J Hawksworth DL Sastry KN (2006) *Vittatispora*, a new melanosporaceous genus from Indian soil. Mycologia 98(3): 460–467. 10.1080/15572536.2006.11832681 17040075

[B10] Clements FE (1909) Genera of Fungi. H. W. Wilson, Minneapolis, 227 pp.

[B11] Corda ACJ (1837) Icones fungorum hucusque cognitorum. Volume 1. Calve, Prague, 32 pp.

[B12] Corda ACJ (1842) Icones fungorum hucusque cognitorum. Volume 5. Calve, Prague, 92 pp.

[B13] Davey ML Tsuneda A Currah RS (2008) Evidence that the gemmae of *Papulasporasepedonioides* are neotenous perithecia in the Melanosporales. Mycologia 100(4): 626–635. 10.3852/08-001R 18833755

[B14] Dennis RWG (1968) British Ascomycetes. Cramer, Lehre, Germany, 455 pp.

[B15] Dettman JR Harbinski FM Taylor JW (2001) Ascospore morphology is a poor predictor of the phylogenetic relationships of *Neurospora* and *Gelasinospora*. Fungal Genetics and Biology 34(1): 49–61. 10.1006/fgbi.2001.1289 11567551

[B16] Doguet G (1955) Le genre *Melanospora* biologie, morphologie, développement, systématique. Botaniste 39: 1–313.

[B17] Fan L Hou C Cannon PF Li Y (2012) A new species of *Melanospora* on truffles from China. Mycologia 104(6): 1433–1442. 10.3852/11-338 22684289

[B18] Figueras MJ Guarro J (1988) A scanning electron microscopic study of ascoma development in *Chaetomiummalaysiense*. Mycologia 80(3): 298–306. 10.2307/3807625

[B19] Fuckel L (1877) Symbolae mycologicae. Beiträge zur Kenntniss der rheinischen Pilze. Dritter Nachtrag. Jahrbücher des Nassauischen Vereins für Naturkunde 29-30: 1–39.

[B20] García D Stchigel AM Guarro J (2003) A new species of *Poroconiochaeta* from Russian soils. Mycologia 95(3): 525–529. 10.1080/15572536.2004.11833099 21156643

[B21] García D Stchigel AM Cano J Guarro J Hawksworth DL (2004) A synopsis and re-circumscription of *Neurospora* (syn. *Gelasinospora*) based on ultrastructural and 28S rDNA sequence data. Mycological Research 108(10): 1119–1142. 10.1017/S0953756204000218 15535064

[B22] Gaümann E (1964) Die Pilze. Birkhäuser Verlag, Basel, Switzerland, 541 pp 10.1007/978-3-0348-6860-0

[B23] Goh YK Vujanovic V (2010) *Sphaerodesquadrangularis* biotrophic mycoparasitism on *Fusariumavenaceum*. Mycologia 102(4): 757–762. 10.3852/09-171 20648743

[B24] Guarro J Gene J Stchigel AM Figueras MJ (2012) Atlas of Soil Ascomycetes. CBS Biodiversity Series no. 10. CBS-KNAW Fungal Biodiversity Centre, Utrecht, the Netherlands, 486 pp.

[B25] Harveson RM Kimbrough JW (2000) First report of *Persiciosporamoreaui*, a parasite of *Fusariumoxysporum*, in the western hemisphere. Mycotaxon 76: 361–365.

[B26] Harveson RM Kimbrough JW (2001) Parasitism and measurement of damage to *Fusariumoxysporum* by species of *Melanospora*, *Sphaerodes*, and *Persiciospora*. Mycologia 93(2): 249–257. 10.2307/3761645

[B27] Hausner G Reid J (2004) The nuclear small subunit ribosomal genes of *Sphaeronaemellahelvellae*, *Sphaeronaemellafimicola*, *Gabarnaudiabetae*, and *Cornuvesicafalcata*: phylogenetic implications. Canadian Journal of Botany 82(6): 752–762. 10.1139/b04-046

[B28] Hernández-Restrepo M Groenewald JZ Elliott ML Canning G McMillan VE Crous PW (2016) Take-all or nothing. Studies in Mycology 83: 19–48. 10.1016/j.simyco.2016.06.002 27504028 PMC4969266

[B29] Hibbett DS Binder M Bischoff JF Blackwell M Cannon PF Eriksson OE Huhndorf S James T Kirk PM Lücking R Thorsten Lumbsch H Lutzoni F Matheny PB McLaughlin DJ Powell MJ Redhead S Schoch CL Spatafora JW Stalpers JA Vilgalys R Aime MC Aptroot A Bauer R Begerow D Benny GL Castlebury LA Crous PW Dai YC Gams W Geiser DM Griffith GW Gueidan C Hawksworth DL Hestmark G Hosaka K Humber RA Hyde KD Ironside JE Kõljalg U Kurtzman CP Larsson KH Lichtwardt R Longcore J Miadlikowska J Miller A Moncalvo JM Mozley-Standridge S Oberwinkler F Parmasto E Reeb V Rogers JD Roux C Ryvarden L Sampaio JP Schüssler A Sugiyama J Thorn RG Tibell L Untereiner WA Walker C Wang Z Weir A Weiss M White MM Winka K Yao YJ Zhang N (2007) A higher-level phylogenetic classification of the Fungi. Mycological Research 111(5): 509–547. 10.1016/j.mycres.2007.03.004 17572334

[B30] Hotson JW (1912) Cultural studies of fungi producing bulbils and similar propagative bodies. Proceedings of the American Academy of Arts and Sciences 48(8): 227−306. 10.2307/20022828

[B31] Hotson HH (1942) Some species of *Papulaspora* associated with rots of gladiolus bulbs. Mycologia 34(4): 391−399. 10.2307/3754980

[B32] Houbraken J Due M Varga J Meijer M Frisvad JC Samson RA (2007) Polyphasic taxonomy of AspergillussectionUsti Studies in Mycology 59: 107−128. 10.3114/sim.2007.59.12 PMC2275196 18490949

[B33] Jones KG Blackwell M (1998) Phylogenetic analysis of ambrosial species in the genus *Raffaelea* based on 18S rDNA sequences. Mycological Research 102(6): 661–665. 10.1017/S0953756296003437

[B34] Jordan EG Barnett HL (1978) Nutrition and parasitism of *Melanosporazamiae*. Mycologia 70(2): 300–312. 10.2307/3759028

[B35] Kim SH Vujanovic V (2016) Relationship between mycoparasites lifestyles and biocontrol behaviors against *Fusarium* spp. and mycotoxins production. Applied Microbiology and Biotechnology 100(12): 5257–5272. 10.1007/s00253-016-7539-z 27121573

[B36] Kim SH Vujanovic V (2017) Biodegradation and biodetoxification of *Fusarium* mycotoxins by *Sphaerodesmycoparasitica* AMB Express 7: 145. 10.1186/s13568-017-0446-6 PMC5500597 28687037

[B37] Kornerup A Wanscher JH (1984) Methuen handbook of colour. 3^rd^ edn. Eyre Methuen, London, England, 252 pp.

[B38] Kowalski DT (1965) The development and cytology of *Melanosporatiffanii*. Mycologia 57(2): 279–290. 10.2307/3756829

[B39] Krug JC (1988) A new species of *Persiciospora* from African soil. Mycologia 80(3): 414–417. 10.2307/3807643

[B40] Krug JC Jeng RS (1979) *Rhytidospora* and *Pteridiosperma*, gen. nov. (Melanosporaceae). Mycotaxon 10(1): 41–45.

[B41] Li D-W Schultes NP Vossbrinck C (2016) *Olpitrichumsphaerosporum*: a new USA record and phylogenetic placement. Mycotaxon 131(1): 123–133. 10.5248/131.123

[B42] Maharachchikumbura SSN Hyde KD Jones EBG McKenzie EHC Huang S-K Abdel-Wahab MA Daranagama DA Dayarathne M D’souza MJ Goonasekara ID Hongsanan S Jayawardena RS Kirk PM Konta S Liu J-K Liu Z-Y Norphanphoun C Pang K-L Perera RH Senanayake IC Shang Q Shenoy BD Xiao Y Bahkali AH Kang J Somrothipol S Suetrong S Wen T Xu J (2015) Towards a natural classification and backbone tree for Sordariomycetes. Fungal Diversity 72(1): 199–301. 10.1007/s13225-015-0331-z

[B43] Matsushima T (1995) Matsushima Mycological Memoirs No. 8. Matsushima Mycological Memoirs 8: 1–44.

[B44] Miller AN Huhndorf S (2004) Using phylogenetic species recognition to delimit species boundaries within *Lasiosphaeria*. Mycologia 96(5): 1106–1127. 10.1080/15572536.2005.11832909 21148930

[B45] Miller AN Huhndorf S (2005) Multi-gene phylogenies indicate ascomal wall morphology is a better predictor of phylogenetic relationships than ascospore morphology in the Sordariales (Ascomycota, Fungi). Molecular Phylogenetics and Evolution 35(1): 60–75. 10.1016/j.ympev.2005.01.007 15737582

[B46] Nygren K Strandberg R Wallberg A Nabholz B Gustafsson T García D Cano J Guarro J Johannesson H (2011) A comprehensive phylogeny of *Neurospora* reveals a link between reproductive mode and molecular evolution in fungi. Molecular Phylogenetics and Evolution 59(3): 649–63. 10.1016/j.ympev.2011.03.023 21439389

[B47] Rehner SA Samuels GJ (1995) Molecular systematics of the Hypocreales: a teleomorph gene phylogeny and the status of their anamorphs. Canadian Journal of Botany 73(S1): 816–823. 10.1139/b95-327

[B48] Roll-Hansen F (1948) *Melanosporaphaseolii* n.sp. Blyttia 6: 73–76.

[B49] Schultes NP Murtishi B Li DW (2017) Phylogenetic relationships of *Chlamydomyces*, *Harzia*, *Olpitrichum*, and their sexual allies, *Melanospora* and *Sphaerodes*. Fungal Biology 121(10): 890–904. 10.1016/j.funbio.2017.07.004 28889913

[B50] Spatafora JW Blackwell M (1994a) Cladistic analysis of partial ssrDNA sequences among unitunicate perithecial ascomycetes and its implications on the evolution of centrum development. In: Hawksworth DL (Ed.) Ascomycete Systematics: problems and perspectives in the nineties.Plenum Press, New York, 233–241.

[B51] Spatafora JW Blackwell M (1994b) The polyphyletic origins of ophiostomatoid fungi. Mycological Research 98(1): 1–9. 10.1016/S0953-7562(09)80327-4

[B52] Stchigel AM Cano J Guarro J (1999) A new species of *Melanospora* from Easter Island. Mycological Research 103(10): 1305–1308. 10.1017/S095375629900845X

[B53] Stchigel AM Cano J Mac Cormack WP Guarro J (2001) *Antarctomycespsychrotrophicus* gen. et sp. nov., a new ascomycete from Antarctica. Mycological Research 105(3): 377–382. 10.1017/S0953756201003379

[B54] Stchigel AM Guarro J Figueras MJ (1997) A new species of *Melanospora* from India. Mycological Research 101(4): 446–448. 10.1017/S0953756296002948

[B55] Van Beyma JFH (1931) Untersuchungen über Russtaupilze. Verhandelingen Koninklijke Nederlandse Akademie van Wetenschappen Afdeling Natuurkunde 29(2): 1–40.

[B56] Vilgalys R Hester M (1990) Rapid genetic identification and mapping of enzymatically amplified ribosomal DNA from several species of *Cryptococcus* Journal of Bacteriology 172(8): 4238−4246. 10.1128/jb.172.8.4238-4246.1990 PMC213247 2376561

[B57] Voigt K Wöstemeyer J (2000) Reliable amplification of actin genes facilitates deep-level phylogeny. Microbiological Research 155(3): 179–195. 10.1016/S0944-5013(00)80031-2 11061186

[B58] Vujanovic V Goh YK (2009) *Sphaerodesmycoparasitica* sp. nov., a new biotrophic mycopaparasite on *Fusariumavenaceum, F. graminearum* and *F.oxysporum*. Mycological Research 113(10): 1172–1180. 10.1016/j.mycres.2009.07.018 19857813

[B59] White TJ Bruns T Lee S Taylor J (1990) Amplification and direct sequencing of fungal ribosomal RNA genes for phylogenetics. In: Innis MA Gelfand DH Sninsky JS White TJ (Eds) PCR protocol: a guide to methods and applications.Academic Press, San Diego, 315–322. 10.1016/B978-0-12-372180-8.50042-1

[B60] Winter G (1887) Dr. L. Rabenhorst’s Kryptogamen-Flora von Deutschland, Oesterreich und der Schweiz. E. Kummer, Leipzig, Germany, 928 pp.

[B61] Woronichin NN (1924) Fungi nonnulli novi e Caucaso. III. Notulae Systematicae ex Instituto Cryptogamico Horti Botanici Petropolitani 3(2): 31–32.

[B62] Zhang N Blackwell M (2002) Molecular phylogeny of *Melanospora* and similar pyrenomycetous fungi. Mycological Research 106(2): 148–155. 10.1017/S0953756201005354

[B63] Zhang N Castlebury LA Miller AN Huhndorf SM Schoch CL Seifert KA Rossman AY Rogers JD Kohlmeyer J Volkmann-Kohlmeyer B Sung GH (2006) An overview of the systematics of the Sordariomycetes based on a four-gene phylogeny. Mycologia 98(6): 1076–1087. 10.1080/15572536.2006.11832635 17486982

[B64] Zhang M Wang R-L Hu H (2004) Bulbils exist in root of *Cypripediumflavum*. Acta Botanica Yunnanica 26(5): 495–49.

